# Long COVID in Children, Adults, and Vulnerable Populations: A Comprehensive Overview for an Integrated Approach

**DOI:** 10.3390/diseases12050095

**Published:** 2024-05-06

**Authors:** Valeria Calcaterra, Sara Zanelli, Andrea Foppiani, Elvira Verduci, Beatrice Benatti, Roberto Bollina, Francesco Bombaci, Antonio Brucato, Selene Cammarata, Elisa Calabrò, Giovanna Cirnigliaro, Silvia Della Torre, Bernardo Dell’osso, Chiara Moltrasio, Angelo Valerio Marzano, Chiara Nostro, Maurizio Romagnuolo, Lucia Trotta, Valeria Savasi, Valeria Smiroldo, Gianvincenzo Zuccotti

**Affiliations:** 1 Department of Internal Medicine and Therapeutics, Università degli Sudi di Pavia, 27100 Pavia, Italy; valeria.calcaterra@unipv.it; 2 Pediatric Department, Buzzi Children’s Hospital, 20154 Milano, Italy; zanelli.sara01@gmail.com (S.Z.); elvira.verduci@unimi.it (E.V.); 3 International Center for the Assessment of Nutritional Status and the Development of Dietary Intervention Strategies (ICANS-DIS), Department of Food, Environmental and Nutritional Sciences (DeFENS), Università degli Studi di Milano, 20157 Milano, Italy; andrea.foppiani@unimi.it; 4 IRCCS Istituto Auxologico Italiano, Department of Endocrine and Metabolic Medicine, Clinical Nutrition Unit, 20145 Milano, Italy; 5 Department of Health Sciences, Università degli Studi di Milano, 20157 Milano, Italy; 6 “Aldo Ravelli” Center for Nanotechnology and Neurostimulation, Università degli Studi di Milano, 20157 Milano, Italy; beatrice.benatti@asst-fbf-sacco.it (B.B.); bernardo.dellosso@unimi.it (B.D.); 7 Department of Psychiatry, ASST Fatebenefratelli-Sacco, University of Milano, 20154 Milano, Italy; giovanna.cirnigliaro@asst-fbf-sacco.it (G.C.); chiara.nostro@asst-fbf-sacco.it (C.N.); 8 Department of Medical Oncology, ASST Rhodense, 20024 Milano, Italy; rbollina@asst-rhodense.it (R.B.); sdellatorre@asst-rhodense.it (S.D.T.); vsmiroldo@asst-rhodense.it (V.S.); 9 Department of Radiology, ASST Fatebenefratelli Sacco, 20154 Milano, Italy; francesco.bombaci@asst-fbf-sacco.it; 10 Department of Internal Medicine, ASST Fatebenefratelli-Sacco, 20154 Milano, Italy; antonio.brucato@unimi.it (A.B.); elisa.calabro@asst-fbf-sacco.it (E.C.); lucia.trotta@asst-fbf-sacco.it (L.T.); 11 Department of Woman, Mother and Neonate, Luigi Sacco Hospital, ASST Fatebenefratelli-Sacco, 20154 Milano, Italy; selene.cammarata@asst-fbf-sacco.it (S.C.); valeria.savasi@unimi.it (V.S.); 12 Department of Psychiatry and Behavioral Sciences, Stanford University, Stanford, CA 94305, USA; 13 Centro per lo Studio dei Meccanismi Molecolari alla Base delle Patologie Neuro-Psico-Geriatriche, Università degli Studi di Milano, 20157 Milano, Italy; 14 Dermatology Unit, Fondazione IRCCS Ca’ Granda Ospedale Maggiore Policlinico, 20122 Milano, Italy; chiara.moltrasio@policlinico.mi.it (C.M.); angelo.marzano@unimi.it (A.V.M.); maurizio.romagnuolo@unimi.it (M.R.); 15 Department of Pathophysiology and Transplantation, Università degli Studi di Milano, 20122 Milano, Italy; 16 Department of Biomedical and Clinical Science, Università degli Studi di Milano, 20157 Milano, Italy

**Keywords:** long COVID, children, adults, pregnancy, oncological, SARS-CoV-2, post-COVID-19 syndrome, integrated approach

## Abstract

Long COVID affects both children and adults, including subjects who experienced severe, mild, or even asymptomatic SARS-CoV-2 infection. We have provided a comprehensive overview of the incidence, clinical characteristics, risk factors, and outcomes of persistent COVID-19 symptoms in both children and adults, encompassing vulnerable populations, such as pregnant women and oncological patients. Our objective is to emphasize the critical significance of adopting an integrated approach for the early detection and appropriate management of long COVID. The incidence and severity of long COVID symptoms can have a significant impact on the quality of life of patients and the course of disease in the case of pre-existing pathologies. Particularly, in fragile and vulnerable patients, the presence of PASC is related to significantly worse survival, independent from pre-existing vulnerabilities and treatment. It is important try to achieve an early recognition and management. Various mechanisms are implicated, resulting in a wide range of clinical presentations. Understanding the specific mechanisms and risk factors involved in long COVID is crucial for tailoring effective interventions and support strategies. Management approaches involve comprehensive biopsychosocial assessments and treatment of symptoms and comorbidities, such as autonomic dysfunction, as well as multidisciplinary rehabilitation. The overall course of long COVID is one of gradual improvement, with recovery observed in the majority, though not all, of patients. As the research on long-COVID continues to evolve, ongoing studies are likely to shed more light on the intricate relationship between chronic diseases, such as oncological status, cardiovascular diseases, psychiatric disorders, and the persistent effects of SARS-CoV-2 infection. This information could guide healthcare providers, researchers, and policymakers in developing targeted interventions.

## 1. Introduction 

The COVID-19 pandemic has affected millions of lives globally [1]. Nonetheless, treatments have been devised over this period, and successful vaccines have been extensively distributed to the population, encompassing both children and adults, safeguarding millions from severe illness and death [2]. Compared to adults, children contract less frequently Severe Acute Respiratory Syndrome Coronavirus-2 (SARS-CoV-2) infection, and among the infected, children have less severe disease [3].

Although vaccination and improved treatment have significantly impacted on the severity of acute COVID-19 infection, one in 10 people presents a persistence of symptoms known as post-COVID-19 syndrome or long COVID [4].

In October 2021, the World Health Organization (WHO) defined long COVID as the continuation or development of new symptoms 3 months after the initial SARS-CoV-2 infection, with these symptoms lasting for at least 2 months with no other explanation [5]. In February 2022, the National Institute for Health and Care Excellence (NICE) issued guidelines that characterize long COVID as signs and symptoms that persist or emerge following acute COVID-19. This encompasses ongoing symptomatic COVID-19 (lasting from 4 to 12 weeks) and post-COVID-19 syndrome (12 weeks or more) [6].

Long COVID affects both children and adults, including subjects who experienced severe, mild, or even asymptomatic SARS-CoV-2 infection [7]. 

**Figure 1:**
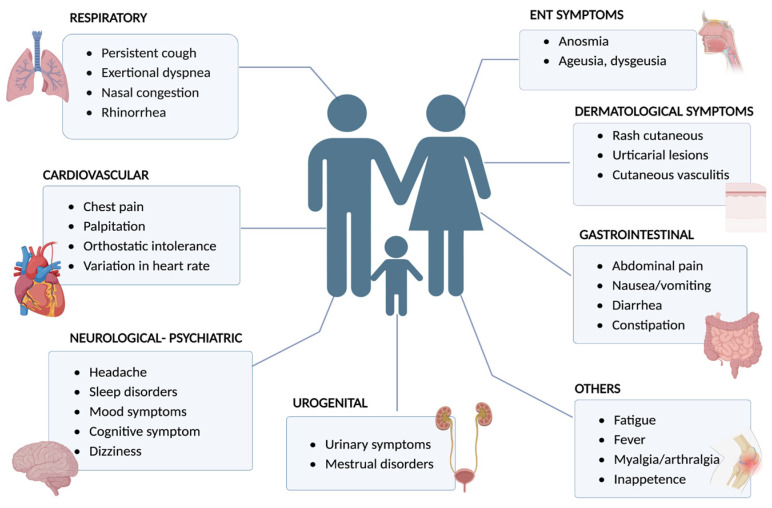
Long COVID symptoms in adults and children (created with Biorender https://www.biorender.com, accessed on 12 March 2024).

The pathogenic mechanisms of long COVID remain not fully understood; moreover, a multifactorial origin, including an exaggerated immune response, viral persistence, cross-reactive autoimmune responses, genetics, drugs, and other factors, has been postulated [8]. Being female, of a younger age, belonging to a Black, mixed ethnicity, or other ethnic minority groups, experiencing socioeconomic deprivation, smoking, a high body mass index (BMI), and the presence of various comorbidities were associated with an increased risk of long COVID [9]. Long COVID is characterized by a multidimensional symptomatology (Figure 1) and disability. The predominant symptoms include fatigue, headache, brain fog, and myalgia. Sequelae were categorized based on the systems or organs involved in respiratory symptoms, residual fatigue, weight loss, neurocognitive sequelae, non-respiratory residual organ dysfunction, and others [10,11]. Long COVID symptoms negatively impact the function, activities, participation, and quality of life of individuals [12]. 

There is no specific treatment for long COVID; nutritional support, lifestyle adjustments, and exercise-based therapy are essential parts of the management of this condition [12]. Considering the diversity of symptoms, the management of patients requires a multidisciplinary team approach [13], and telemedicine, digital, medical internet, and eHealth interventions targeting social support have been considered essential for addressing the long-term negative effects [14].

The early recognition of patients with post-COVID-19 syndrome is essential to undertake a complete and effective diagnostic–therapeutic approach and to guarantee adequate care. Long-term monitoring is mandatory to define the prevalence of complications post-infection and to understand whether the disease can be considered a long-term risk condition for health.

In the context of a national project on the integrated approach to post-COVID-19 syndrome, we provided a comprehensive overview of the incidence, clinical characteristics, risk factors, and outcomes of persistent COVID-19 symptoms in both children and adults, encompassing vulnerable populations, such as pregnant women and oncological patients. Our objective is to emphasize the critical significance of adopting an integrated approach for the early detection and appropriate management of long COVID.

## 2. Methods 

We conducted a narrative review examining the multisystemic effects of long COVID in both children and adults, with a focus on its incidence, clinical characteristics, risk factors, and outcomes. Our comprehensive, non-systematic literature search was conducted on PubMed/MEDLINE databases, with a language restriction to English. 

The inclusion criteria were: (a) publication date: from January 2020 to February 2024; (b) age: children (0–18 years old) and adults (>18 years old); and (c) type of article: reviews, meta-analyses, prospective, retrospective, cross-sectional cohort studies, case–control studies, clinical practice guidelines, and editorials. Single case reports without a literature review and articles published as abstracts and non-English-language papers were excluded. 

For the study, we categorized publications by pediatric and adult age groups, organizing them based on different organs or systems. The keyword list used for the research, categorized according to the target population/organ, is detailed in Table 1; the number of potentially relevant manuscripts and the number of considered manuscripts resulting from the literature search are also reported.

The term “long COVID” was considered as symptoms persisting beyond the acute phase of COVID-19; all established definitions of long COVID are permissible, including proprietary definitions proposed by the study’s authors.

**Figure 2:**
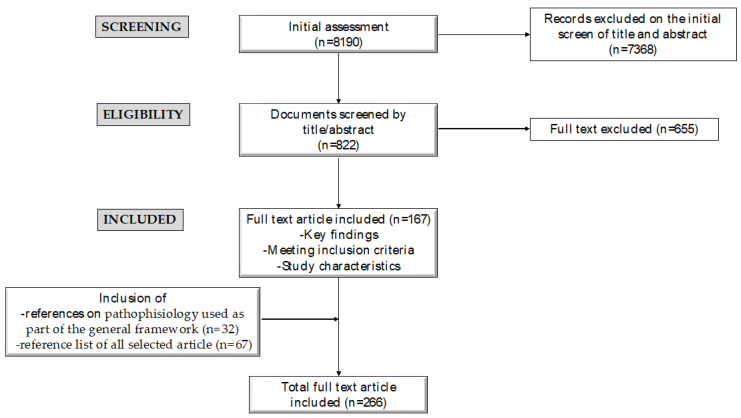
Flowchart of the selection process for studies and relevant articles considered.

From an initial selection of 8190 papers screened by title/abstract, we proceeded to evaluate the full texts of potentially relevant articles (*n* = 822). The authors then reviewed, analyzed, and discussed the full texts of the relevant papers (*n* = 167). The reference lists of all manuscripts were also scrutinized to identify additional pertinent studies. Additionally, pertinent studies discussing pathophysiology and used as part of the general framework of the topic were also considered. The flowchart outlining the selection process for studies and relevant articles considered is schematically depicted in Figure 2. 

The resulting draft underwent a through discussion with all co-authors and received unanimous approval.

## 3. Pathophysiology of Long COVID

The pathophysiology of long COVID is still unclear, but there are several hypotheses about it. The gateway used by SARS-CoV-2 to invade cells is the Angiotensin Converting Enzyme-2 (ACE-2) receptor. This is expressed at the epithelium of many organs and thus can explain the variety of symptoms of long COVID syndrome [15]. A number of factors can be involved in pathophysiology: chronic organ damage, SARS-CoV-2 persistence in the organism, dysregulation of the immune system, and thus the persistence of chronic inflammation and autoimmunity. Moreover, lengthy organ recovery time, effects of hospitalization, sequelae of critical illness, post-intensive care syndrome, complications arising from comorbidities, and adverse medication effects could be implicated [16]. 

Viral persistence several months after acute infection seems to be a potential mechanism implicated in pathogenesis because of the resulting pathologic inflammation [17]. Prolonged exposure to SARS-CoV-2 or its components could indeed exacerbate the immune response with increased levels of proinflammatory cytokines, such as IL-6, IL-1β, and TNF, and these can cause organ damage [18].

As in autoimmune diseases, the dysfunction of T cells can promote long COVID through autoreactive lymphocytes [19]. Furthermore B cells are involved in pathogenesis; indeed, in COVID-19 patients, several autoantibodies, such as antiphospholipid autoantibodies [20], can be detected, but also autoantibodies against interferons, neutrophils, connective tissues, cyclic citrullinated peptides, and cell nuclei, were identified in COVID-19 patients [21]. These may contribute to typical symptoms of this syndrome, like fatigue, headache, concentration difficulties, and joint pain. Additionally, lymphopenia and hyperinflammation may contribute to long COVID and the chronic immune activation typical of this syndrome [22,23].

Moreover, gut microbiota remain significantly altered after recovery from COVID-19 [24,25], and microbiota dysbiosis is correlated with increased inflammatory biomarkers, which may contribute to persistent symptoms, in particular, gastrointestinal but also neurological ones, given the role of the gut–brain axis in the control of central nervous system inflammation [17].

There is a reduced ability to clear SARS-CoV-2 because of a diminished immunologic capacity, and so the persistence of the virus seems to have a genetic basis. The result is excessive proinflammatory cytokine production, enhanced by the production of reactive oxygen species leading to typical long COVID symptomatology [17]. This process can complement platelet activation and encourage impaired coagulation, enhancing the risk of microthrombosis in multiple organs [15]. A possible role in the pathogenesis of long COVID is attributed to perivascular inflammation and thromboembolism [26]. More studies are necessary to investigate a possible pro-fibrotic state that is already observed in adult patients.

The evidence concerning long COVID, variants of SARS-CoV-2, and their role in pathogenesis is limited. Many studies have explored the contribution of genetic factors associated with severe COVID-19, such as blood group, epigenomic markers, and traits associated with protection, but the potential contribution of genetic factors to long COVID is still unexplored [18].

## 4. Long COVID in Pediatric-Age Patients 

Acute SARS-CoV-2 infection is generally mild or asymptomatic in children and adolescents with a low hospitalization and mortality rate [27,28]. The persistence of symptoms has been observed in some patients previously infected with SARS-CoV-2, formerly in adults, and then in the pediatric population [11]. Long COVID syndrome or Post-Acute Sequelae of COVID-19 Syndrome (PASC) in the pediatric-age population was described firstly in 2020 by Ludvigsonn et al. in a small group of Swedish children [29], and other authors reported the presence of PASC in a larger cohort of Australian pediatric COVID-19 patients [30]. Similarly, an Italian cross-sectional study reported persistent symptoms in children previously diagnosed with COVID-19 [31]. 

Long COVID is a multisystemic condition with heterogeneous manifestations involving cardiovascular, respiratory, neurological, and gastrointestinal systems. While the symptoms are mostly mild, they still have a significant impact on the daily functioning, quality of life, and mental health of the pediatric population. Moreover, distinguishing functional complaints from post-acute COVID-19 from those caused by social restrictions remains challenging [32].

Overall, the prevalence of long COVID in children varies from 4% to 66%, depending on the studies [15,33,34]. This wide variability can be attributed to the heterogeneous characteristics of the selected studies: the different sample sizes considered, varying follow-up durations, and different assessment modalities (e.g., telephone survey or clinical examinations), as well as differences in the age of enrolled patients. Lopez-Leon et al. described in a meta-analysis and systematic review the prevalence of long COVID in 25.24% of children previously affected by COVID-19, while the prevalence in hospitalized patients was 29.19% [2]. In a pediatric cohort, Bloise et al. reported that the prevalence of long-lasting symptoms was around 20% [35], while Miller et al. described a prevalence of 2.6% in a cohort study conducted in England and Wales [36]. In an Italian study on 129 children diagnosed with COVID-19, 42.6% of those patients presented at least one symptom over 60 days after infection [31]. Calcaterra et al. conducted a prospective study involving children who were hospitalized and followed up at 3, 6, and 12 months after discharge [37]. The study showed that 16.5% of the patients reported experiencing at least one symptom indicative of long COVID; these symptoms included weight loss (31.6%), inappetence (26.3%), chronic cough (21.1%), fatigue (21.1%), as well as sleep disturbances, wheezing, abdominal pain, and mood disorders (15.8%).

### 4.1. Risk Factors for Long COVID in Children

There are several risk factors associated with long COVID in children and adolescents: older age, female gender, severe COVID-19, comorbidities such as overweight and obesity, allergic diseases, and other chronic disorders [2,38]. 

In a prospective study conducted on 90 children after acute SARS-CoV-2 infection, several symptoms were more common among older children [39]. Borch et al. demonstrate in their study that the age distribution of symptoms differs, with older school children being more frequently affected compared to younger school and pre-school children [40].

As for gender, in a cross-sectional study by Berg et al., female children were more prone to manifest persistent symptoms than males [41]. Long COVID symptoms are more frequent among female patients, according to Roge et al.’s cohort study [42]. Other studies do not highlight any gender differences in pediatric-age patients [43].

As for ethnicity, Rayner et al., in a meta-analysis, identify that the Black race may be associated with a decreased risk of long COVID in children and adolescents [44], but further research is necessary.

The specific SARS-CoV-2 variant is probably another important factor to be considered. The prevalence of pediatric long COVID should have progressively decreased with the Omicron variant’s emergence [45]. An Italian prospective study reports in 15/115 patients long-lasting symptoms, but only four of them were identified with a specific SARS-CoV-2 variant: three were associated with the Omicron variant and one was linked to the Delta variant [37]. The prevalence of long COVID seems to be lower in patients infected with Alpha, Delta, and Omicron variants rather than the ancestral one [46]. In a study, the Delta variant showed a higher viral load in the respiratory tract of adult patients, and it could have also had an impact on children and adolescents [47]. Limited evidence exists about the correlation between symptoms related to long COVID in children and its variants. 

In adult patients, obesity or overweight is described as a long COVID risk factor, and recently it was also identified by Bloise et al. in pediatric patients [32,35]. Allergic diseases and long-term conditions could be possible risk factors [48]. Furthermore, respiratory diseases, asthma, and heart disease are risk factors for long COVID in the pediatric population [44]. 

The severity of acute infection and symptom duration have no correlation according to some studies [31], but other studies report that severity is correlated with the frequency and duration of sequelae [49]. Children with asymptomatic or paucisymptomatic COVID-19 could be affected and develop persisting symptoms [31]. An association between longer hospitalization and more severe persistent symptoms has been reported [33].

The use of COVID-19 vaccines, reducing the incidence and severity of SARS-CoV-2 infection, may also reduce the risk of long COVID [50]; conversely, patients with long COVID have no significant improvements of their symptoms after vaccination [51]. A recent, large, retrospective cohort study has shown a moderate protective effect of vaccinations against this syndrome that is stronger in adolescents and wanes over time [52]. 

The role of genetics is still unclear in the post-COVID-19 condition [18], while studies have verified the existence of genetic features connected to the high susceptibility and severity of acute infection [53,54].

### 4.2. Clinical Manifestation of Long COVID in Pediatric-Age Individuals

COVID-19 in children often presents as asymptomatic or with few symptoms. Hospitalization rates are lower compared to adult patients, and life-threatening complications are infrequent [15]. 

A long-term consequence of COVID-19 is long COVID, characterized by highly variable intensity and duration. Signs and symptoms in pediatric cases are mostly mild and closely resemble those described for adults [55]. They can occur alone or in combination, be intermittent or transient, and may change over time or remain constant. Hospitalization for long COVID symptoms is not usually reported [32].

Long COVID is characterized by multiorgan involvement and highly variable intensity and duration. Symptoms in the pediatric-age population are very similar to those described for adults.

In a meta-analysis by Lopez-Leon et al., mood symptoms are described as the most common (16.5%), in particular, anxiety, depression, sadness, anger, and tension. Furthermore, fatigue (9.66%) and sleep disorders (8.42%), like insomnia, hypersomnia, and poor sleep quality, are also prevalent. Other frequent symptoms are headaches (7.84%), respiratory symptoms (7.62%), sputum production or nasal congestion (7.53%), cognitive symptoms (especially reduced concentration, learning difficulties, confusion, and memory loss) (6.27%), inappetence (6.07%), exercise intolerance (5.73%), and altered smell (5.60%), which includes hyposmia, anosmia, hyperosmia, parosmia, and phantom smell [2]. 

Gastrointestinal symptoms are possible, but with a low prevalence (<5%). Among these, abdominal pain is the most frequent manifestation, followed by constipation, chronic diarrhea, nausea/vomiting, and dysphagia [2,56]. Furthermore, functional gastrointestinal disorders, such as irritable bowel syndrome (IBS) and altered bowel motility, are reported for long COVID syndrome, but the frequency is still not clarified [57,58]. Gut microbiota composition is significantly altered in patients with COVID-19 [7]. Dysbiosis is persistent, lasting for at least 14 months [59] because the bowel is a long-term reservoir, and it could play a central role in the maintenance of symptoms. Lately, the authors also depicted two rare liver manifestations of long COVID: acute liver injury and hepatitis with cholestasis [60].

Cardiovascular manifestations of long COVID in children in order of frequency are orthostatic intolerance [61], exercise intolerance, chest pain, and a variation in heart rate, while palpitations are less common [2,61,62,63]. 

Exertional dyspnea, a persistent cough, and exercise intolerance are the most common respiratory symptoms in children post-SARS-CoV-2 infection [63]. Esmaeilzadeh et al. reported a persistent cough and asthma-like symptoms with a prevalence of 41.5% in a cohort of COVID-19 hospitalized children [64]. While spirometry and plethysmography are usually normal, abnormality in the six-minute walking test (6MWT) could reveal exercise intolerance in children affected by long COVID [63]. Even months after acute infection, irregular pleural lines, B lines, and subpleural consolidations were detected through lung ultrasounds (LUSs), respectively, in 27.5%, 16.9%, and 8.6% of the cases. Patients could have ultrasound artifacts and show an improvement with the passage of time from acute infection [65]. 

Ear, nose, and throat (ENT) symptoms are possible long COVID presentations at a pediatric age. A sore throat was described in 2.47% of patients, whilst dysphonia was uncommon [2]. Earache, tinnitus, and vertigo may be present with a prevalence around 3%, as Lopez-Leon et al. described in their meta-analysis. Buonsenso et al. reported nasal congestion in 12.4% of children with persistent symptoms [31], and in a cohort study, Roge et al. demonstrated the presence of rhinorrhea in 16.1% of pediatric patients [42]. Olfactory dysfunction, such as anosmia or dysgeusia, are described in 12.3% of children with post-COVID-19 syndrome [42]. However, in a prospective cohort study, Osmanov et al. described a more rapid improvement of smell disturbances than other symptoms of long COVID [48].

Fatigue and chronic fatigue syndrome (CFS) are common symptoms in children affected by long COVID [66,67]. In an English CLoCK study, fatigue, in addition to concentration problems and headaches, was the most frequent symptom in the case group [28]. In an Italian survey carried out by Buonsenso et al. on 510 children after contracting COVID-19, their parents reported an impairment in physical activity due to fatigue, and only 51 patients returned to prior levels of activity [17]. Furthermore, the more physically active the children were before infection, the more likely they returned to that level. CFS is associated with a number of viral infections, including SARS-CoV-2, and so it has acquired high epidemiological relevance [67].

Balance problems, like dizziness, are described in children and adolescents, but merely with a prevalence of 4.4%. Arthralgia and myalgia are also common symptoms, and Lopez-Leon et al. indicate a prevalence of 3.76% in pediatric patients [2].

About the dermatological symptoms, skin rashes are a possible manifestation of this condition, with a prevalence of 2.61% [2].

During the COVID-19 pandemic, the isolation, loss of parents or caregivers, increased stress, and a fear of COVID-19 had a significant impact on children’s development [68]. A new onset of psychological problems, like anxiety, depression, irritability, boredom, and inattention, were displayed by children during this pandemic. Children with behavioral problems, like autism and attention deficit and hyperactivity disorder (ADHD), have a high probability for worsening symptoms [69]. 

In an Italian study, parents reported a wide range of neurocognitive symptoms: lack of concentration, difficulty processing/remembering information or understanding instructions, short-term memory issues, and struggling to find the right words [70]. As in adult patients, visuospatial abilities and executive function are the most often affected [71]. In the Danish LongCOVIDKidsDK study, headaches and concentration difficulties are the most frequent manifestation in the case group [41]. It is important to highlight the ongoing development of the nervous system of children and the differential expression of cell receptor targets over time with a variable susceptibility to neurological alteration in post-COVID-19 syndrome [72].

Long COVID symptoms usually disappear spontaneously, leading to a good prognosis [55]. In most patients, long COVID symptoms resolved in the range of 1–5 months in a minimum of 54–75% of children [40]. In a prospective study, Molteni et al. report a symptomatic illness lasting 4 weeks or longer in only 4.4% of patients and lasting 8 weeks or longer in just 1.8% of cases [73]. 

A follow-up study between 4 and 12 weeks after acute SARS-CoV-2 infection is essential in pediatric cases, particularly in high-risk patients, such as those with asthma, allergic diseases, obesity, neuropsychiatric disorders, and/or other comorbidities, including those who are asymptomatic. Tailored investigations are necessary due to the significant impact of long COVID on children’s daily lives, especially at home and in school. A multidisciplinary team is essential due to the involvement of multiple organs. Currently, there are no dedicated guidelines for treating long COVID in children. Physical rehabilitation, symptomatic medication (e.g., paracetamol), and, if necessary, psychological support could be beneficial to these patients. On the other hand, vaccination in children and adolescents is essential to protect them from the long-term consequences of acute COVID-19. 

In Table 2, a list of the main studies on long COVID on pediatric patients is presented.

## 5. Long COVID in Adults

### 5.1. Risk Factors for Long COVID in Adults

Several risk factors are associated with long COVID in adults. First of all, sociodemographic characteristics are involved. According to many studies, the female sex is more frequently affected by persistent symptoms after SARS-CoV-2 acute infection [9,23]. In a review, Koch et al. state that female patients are five-times more likely to develop long COVID symptoms after discharge than males [74]. Furthermore, an Italian prospective cohort study on 377enrolled patients revealed a higher risk of being diagnosed with long COVID in females [75]. 

Other significant risk factors include a White ethnicity, which is associated with an elevated risk of disease [76], and advanced age. Older patients appear to have a higher likelihood of developing long COVID compared to younger individuals [74,77]. However, it is important to note that older patients often have pre-existing comorbidities and may experience a more severe acute infection, which could further increase their risk.

Both current smokers and former smokers also have a higher risk of experiencing long COVID compared to those who have never smoked [9]. Given that smokers typically have compromised immune and cardiovascular systems, smoking may potentially contribute to the persistence of symptoms [78].

Pre-existing comorbidities serve as significant risk factors as well: conditions such as hypertension, diabetes, chronic kidney disease, cerebrovascular disease, chronic obstructive pulmonary disease (COPD), or cardiovascular disease may contribute to the onset of long COVID [77]. Additionally, obesity and overweight are associated with a high disease risk. A meta-analysis by Tsampasian et al. confirmed that a higher BMI is associated with a higher risk of developing long COVID [79]. Concerning antecedent allergic diseases, in a systematic review, Wolff et al. reported individuals with asthma and rhinitis may have a higher risk of long COVID [80]. Lastly, poor mental health (e.g., anxiety and depression) may also be a significant risk factor [76].

The severity of acute SARS-CoV-2 infection is also associated with long COVID. Tsampasian et al., in a recent meta-analysis, report that ICU admission and hospitalization, which are indicative of severe disease, are associated with long COVID [79]. Even in a review by Su et al., critical illness, acute respiratory syndrome, long-term ventilator support, and multiorgan impairment caused by COVID-19 are considered as risk factors for post-acute COVID-19 symptoms [81]. Although this syndrome is also described in non-hospitalized patients with mild acute infections [75], the frequency is lower than in severe cases.

Chronic diseases, such as diabetes, asthma, and cardiovascular diseases, appear to have a bidirectional relationship with COVID-19. These comorbidities, prevalent in the adult population, particularly the elderly, serve as risk factors for both contracting COVID-19 and experiencing prolonged symptoms following acute SARS-CoV-2 infection. Furthermore, COVID-19 may exacerbate these pre-existing chronic conditions [82,83,84].

Data on the prevalence of long COVID in specific chronic disorders remain limited. Heald et al. found that long COVID was more prevalent in men with type 2 diabetes (T2D) than in matched non-T2D controls, while the opposite trend was observed for women with T2D, with similar rates of recorded long COVID for both T2D men and women [85]. Sharif detected a co-prevalence of cardiovascular diseases, diabetes, and long COVID among 11.9% of patients [86]. Even though Hung et al. described a higher prevalence of long COVID in adults with asthma compared to the general population [87], the published data neither confirm nor deny whether long-term COVID-19 symptoms in patients with an asthma diagnosis differ in strength and frequency from those without an asthma diagnosis [88]. 

Additionally, certain specific populations have been identified as high-risk patients. Pregnant women, for instance, are considered to be at a heightened risk compared to the non-obstetric population. This is evidenced by an increased incidence of clinical manifestations, severity of symptoms, and associated obstetric morbidity [9].

Similarly, oncology patients have been shown to be more susceptible to acute and severe COVID-19 infection [89], with a fatality rate estimated at 22.4% among COVID-19 patients with cancer, as opposed to 5.9% in patients without cancer. The systemic immunosuppressive status of cancer patients, whether caused by the disease itself or anticancer treatment, confers an increased risk of COVID-19 [90]. Simultaneously, the incidence of post-acute sequelae of SARS-CoV-2 infection, known as COVID-19 long-term effects, has been described in oncological studies. These studies identified a subgroup of patients with significantly worse survival rates. Particularly, patients who experienced one or more COVID-19 sequelae had significantly shorter post-COVID-19 survival compared to those with a complete resolution of symptoms [47]. 

SARS-CoV-2 variants appear to play a role in determining the risk of long COVID. The prevalence of long COVID is higher in individuals infected with the historical strain than posterior variants (e.g., Alpha, Delta, and Omicron) [46]. In particular, the risk seems to have decreased with the spread of the Omicron variant [81]. 

### 5.2. Clinical Manifestation of Long COVID in Adults

#### 5.2.1. Cutaneous Manifestations 

Since the beginning of the pandemic, the cutaneous manifestations of COVID-19 have been categorized into six main phenotypes: (1) urticarial rash; (2) confluent erythematous/maculopapular/morbilliform rash; (3) papulo-vesicular exanthem; (4) chilblain-like acral pattern; (5) livedo reticularis/racemosa-like pattern; and (6) purpuric vasculitic pattern [91]. However, there have been several reports of a miscellany of other cutaneous presentations related to COVID-19 that cannot be included in this classification [92,93,94,95,96]. To date, confluent erythematous/maculopapular/morbilliform rashes, urticarial lesions, cutaneous vasculitis and vasculopathies, and a chilblain-like acral pattern are the most frequently reported COVID-19-associated cutaneous manifestations [97,98]. 

The pathogenic mechanisms remain incompletely understood, but an immune-mediated inflammatory response, rather than a direct virus-induced cytopathic effect, has been suggested [99,100]. Additionally, an ineffective immune response to the virus or elevated viral loads could be risk factors for the cutaneous transmission of SARS-CoV-2 [99].

The landscape of cutaneous presentations Is likely to evolve due to novel variants of the virus, as demonstrated for the SARS-CoV-2 Omicron variant [101], and long COVID syndrome [102]. The published evidence suggests that long COVID patients, even those who experienced less severe or even asymptomatic diseases, may have a prolonged inflammatory response [103], probably coupled with viral reactivation and/or immune dysregulation able to prevent the resolution of cutaneous manifestations [104]. Recently, using a murine model, Hussain et al. reported a marked absence of hair follicles, the destruction of adipose tissues, and the obliteration of the epidermal layer as long-term post-COVID-19 cutaneous effects [105]. 

The complete destruction of hair follicles is thought to be attributable to several factors, including the direct effect of the virus, the influence of Transforming Growth Factor-β (TGF-β) [106], and changes in the androgen receptor content of dermal papilla [107]. It is noteworthy that Hussain and colleagues [105] demonstrated that treatment with a newly identified 15-amino-acid synthetic peptide, SPIKENET (SPK), which can prevent Spike glycoprotein-1 from binding with host receptors and promote an anti-inflammatory response, successfully restored the above-mentioned long COVID dermatologic changes. This finding suggests that SPK may be considered as a potential therapeutic strategy to prevent long-term cutaneous alterations caused by SARS-CoV-2, thus ameliorating skin disease progression related to COVID-19. 

McMahon et al. [108], through an international registry for COVID-19 dermatological manifestations, evaluated the duration of dermatological signs and symptoms of COVID-19 defining “long haulers” [109] as patients with cutaneous presentations persisting for more than 60 days. In accordance with the UK NICE guidelines [6], only one out of 234 patients included in this registry can now be considered to have had long COVID, experiencing a chilblain-like acral and livedo reticularis pattern for over 22 weeks [108]. Subsequently, Tammaro et al. [110] reported a group of adult patients, including both hospitalized and non-hospitalized individuals, with cutaneous lesions (mainly a confluent erythematous/maculopapular/morbilliform rash and papulovesicular exanthem) lasting for more than 26 weeks after the acute peak of COVID-19. Similarly, Förster et al. [111] reported long-term (more than 12 weeks) cutaneous sequelae in 15 out of 127 hospitalized patients and in 26 out of 588 non-hospitalized subjects. In a Chinese post-acute COVID-19 study of hospitalized patients conducted by Huang et al. [112], only 47 out of 1655 patients (3%) reported skin rashes 26 weeks after disease onset. On the other hand, significant hair loss mainly corresponding to telogen effluvium, secondary to viral infection and/or to emotional and physical stress induced by the disease, was a post-discharge persistent symptom reported in 24 of 120 patients (20%), 110 days after hospital discharge [113].

Interestingly, in a recent study conducted by Grieco et al. [114], the authors investigated cutaneous subjective neurologic symptoms (sNSs) in both subjects with acute COVID-19 and long COVID syndrome. Six long COVID patients suffering from cutaneous sNSs, evaluated through the Neuropathy Total Symptom Score-6 (NTSS-6) questionnaire, underwent a histopathological examination of cutaneous areas affected by paroxysmal diffuse burning and itching sensations. It has been hypothesized that the latter, related to COVID-19 and long COVID as well, may be associated with damage to the cutaneous neurosensory system with symptoms persisting for months and accompanied by systemic manifestations, including, among others, asthenia. In the above-mentioned study, histopathological analyses showed the hypertrophy of dermal nerve fibers resembling dermal hyperneury, a rare form of small nerve hypertrophy that affects sensory C fibers [115], and these findings could explain the dysesthesia experienced by the studied patients. In addition, electroneurography was also performed on two patients, revealing altered dermal A delta and C neuronal fibers, although it remains to be clarified whether this was due to the direct action of the virus on the terminal nerve fibers or other indirect mechanisms [114].

It can be argued that there is a growing need to reveal the exact mechanisms underlying the persistent inflammatory response in cutaneous long COVID patients. To date, there is no specific pattern in the acute phase of COVID-19 able to predict the persistence of long-term lesions or progression to a chronic skin disease. Further investigation is needed to assess the full clinical spectrum, mechanism, and prognostic significance of such cutaneous lesions.

In Table 3, the main selected studies on cutaneous symptoms in adult patients with long COVID are reported.

#### 5.2.2. Gastrointestinal Manifestations 

Long COVID, characterized by a constellation of persistent symptoms lasting months after acute COVID-19 infection, is increasingly recognized. Gastrointestinal (GI) complications are prevalent in long COVID patients, significantly impacting patient quality of life. 

In a systematic review, Choudhury et al. report, in around 12% of long COVID patients, the presence of gastrointestinal symptoms [117]. These manifestations could also occur in adults who experience mild COVID-19; therefore, they are not related to the severity of acute infection. There is no association between gastrointestinal manifestations in long COVID and ethnicity or sex [179].

There are four potential mechanisms that link the SARS-CoV-2 infection to the late development of GI symptoms. Firstly, there may be a direct viral impact, as SARS-CoV-2 may infect GI epithelial cells, leading to inflammation and tissue damage [116]. Secondly, viral infection and immune response may alter the gut microbiome [118,120,122,123,126,127], possibly contributing to other long COVID manifestations, such as chronic fatigue. Thirdly, the potential consequences of COVID-19-induced systemic inflammation are microvascular injury, affecting blood flow and function in the GI tract [116]. Additionally, damage to the nerves controlling gut function can lead to motility issues and digestive problems [116,118,123,126,127]. Finally, the persistent shedding of virions from the GI tract is not excluded [180].

The most common GI manifestations include a loss of appetite, diarrhea, abdominal pain, nausea and vomiting, dyspepsia, and irritable bowel syndrome (IBS) [116,117]. 

Loss of appetite is frequently present, with the prevalence ranging from 26% to 66.7%, depending on studies and populations, and contributing to weight loss and nutritional deficiencies [121,124,126,127]. 

Diarrhea is also recurrently reported (10.4–41.2%); it can be persistent or episodic, impacting hydration and electrolyte balance [118,119,120,124,126,127]. Constipation has also been reported in some cases, occurring in approximately 6.8% of individuals [181].

Abdominal pain (6.7–41%) can be localized or diffuse, often linked to altered gut motility [120,121,124,125,126,127]. 

Nausea and vomiting are also reported (7.7–61.5%), with a significant impact on daily life [118,119,120,124,126,127]. 

Dyspepsia is reported in fewer cases, suggesting potential changes in gastric function, although at least one study did not find significant differences in prevalence for control subjects [118].

Finally, IBS is also present at a higher prevalence compared to the general population (0.3 vs. 3.2%), suggesting a possible link to COVID-19-induced gut dysbiosis [118]. 

Researchers found that gut dysbiosis persists for months, also after SARS-CoV-2 clearance, and contributes to long-term symptoms [182]. In particular, the depletion of commensal anti-inflammatory gut bacteria was observed, which correlated with an increase in the C-reactive protein and, consequently, a pro-inflammatory state [179].

Specifically for GI manifestations, management strategies range from: dietary modifications, with personalized dietary adjustments considering specific symptoms and nutritional needs; probiotics, which may help restore gut microbiota balance and improve symptoms like diarrhea and bloating; medications, specific medications targeting symptoms like diarrhea, constipation, or acid reflux; psychological support, stress management, and anxiety reduction techniques can positively impact gut function; and emerging therapies, exploring the potential of fecal microbiota transplantation and neuromodulation for complex cases.

The research priorities regarding the involvement of the GI tract and long COVID are geared toward understanding the precise mechanisms of GI complications, identifying risk factors, and developing targeted therapies. In particular, there is a need for the individualized management of the symptoms arising, tailoring treatment approaches based on specific patient profiles, and symptom presentations.

In Table 3, the main selected studies on GI symptoms in adult patients with long COVID are reported.

#### 5.2.3. Neurological and Neuropsychiatric Issues 

Neurological and neuropsychiatric symptoms are commonly present in long COVID patients and can last for weeks, even months, after recovery [157]. 

Neurologically, long COVID is marked by a wide spectrum of symptoms [183,184]. COVID-19 patients frequently exhibit a range of neurological complaints: fatigue, cognitive impairment (brain fog, loss of concentration, or memory issues), headache, sleep disturbances, peripheral neuropathy symptoms (pins and needles, and numbness), dizziness, orthostatic impairment, delirium (especially in the elderly), mobility impairment, visual disturbances, mood disorders (depression or anxiety), post-traumatic stress disorder (PTSD), and sensory deficits, like a loss of smell and taste [10,129,130,132,133,135,136,138,141,142,145,148,149,151,152,154,183,185,186]. Moreover, COVID-19 is associated with an elevated risk of developing neurological conditions, including Guillain–Barré syndrome, and neurodegenerative diseases, such as Alzheimer’s [184,187,188].

Neurological and neuropsychiatric complications have varied pathophysiologies, encompassing immune dysregulation, glial cell activation leading to neuronal damage, inflammation, microvascular thrombosis, iatrogenic effects, and psychosocial impacts of the infection [11,78,153]. 

Apart from respiratory symptoms, fatigue and neuropsychiatric symptoms have been the most frequently reported manifestations of long COVID [78].

Within the symptomatology of long COVID, psychiatric disturbances, along with asthenia and intolerance to make an effort, play a primary role in terms of frequency and the impact on daily life, but also in the professional and familiar dimension [139]. Moreover, patients experience a heavy sense of stigma, not achieving a clear diagnosis, having difficulties to access specialists’ services, presenting inconsistent criteria for advanced diagnostic medical examinations, receiving several referrals to other specialists. As a consequence, a considerable degree of emotional feelings, such as anger, frustration, fear, and hopelessness, are documented among these patients [128].

The prevalence of psychiatric symptoms following SARS-CoV-2 infection varies significantly in different studies. Indeed, a study conducted in Italy investigated the prevalence of anxiety and depression in individuals with long COVID, obtaining results ranging from 10.4% to 42% [144]. Another study conducted in Wuhan, China, reported a prevalence of anxiety, depression, and sleep disturbances of about 25%, six months after the resolution of acute illness [112]. Finally, a large-scale study in the United States, on 62,354 subjects [153], estimated an incidence of psychiatric disorders at 18.1% [156]. 

Notably, the prevalence of depression, anxiety, cognitive impairment, PTSD, and sleep disturbances was much higher among long COVID patients than in the general population [131,140]. Interestingly, all these neuropsychiatric manifestations are more frequent in the long term (six months or more after infection) rather than in the first three and six months after acute infection [143,147].

A large number of studies have demonstrated that depression and anxiety are the most commonly reported psychiatric outcomes among SARS-CoV-2 patients [146,155], especially among females [120,157]. 

Among the psychiatric symptoms of long COVID syndrome, the most frequent can be subdivided into five clusters. 

(1) Depression–anxiety. The severity and duration of the acute phase may correlate with the development of depressive and anxious symptoms. Other risk factors for the development of long-term anxiety–depressive disorders include female gender, the presence of pre-existing psychiatric disorders, and the persistence of symptoms attributable to this area one month after infection [150,158]. During the pandemic, females tended to have symptoms of hyperreactivity and negative cognitive and mood disturbances, which consequently could lead to depression onset [157].

(2) PTSD. Risk factors for the development of PTSD include a more severe SARS-CoV-2 infection, especially with the need for intensive care unit admission, female gender, and a pre-existing diagnosis of anxiety–depressive disorder [150].

(3) Cognitive disorders. This category includes concentration disorders, short-term and general memory disorders, reduced attention capacity, language disorders in terms of fluency and encoding, coordination deficits, and signs or symptoms of dementia (according to the ICD-10 diagnostic criteria). Risk factors include advanced age, the presence of pre-existing comorbidities, and high severity and duration of the acute phase of COVID-19 [134,150,158].

(4) Fatigue. This is a condition defined as a reduction in physical and psychological performances generated by central, psychological, or peripheral changes caused by SARS-CoV-2 infection. This manifestation often occurs in the acute phase and can persist in the long term. Risk factors for this condition include female gender and a pre-existing psychiatric diagnosis [150].

(5) Sleep disturbances. Sleep alterations seem to be independent of the severity of COVID-19 infection in the acute phase, which instead seems to be correlated with sleep disturbances that emerge during follow up. Another risk factor for their development is the female gender [150]. While the duration of sleep disturbances following COVID-19 remains uncertain, a study revealed that 10% of patients hospitalized for COVID-19 experienced poor sleep quality, even 12 months after discharge. The link between long COVID and circadian rhythm disorders has also been elucidated. A retrospective cohort study in Ukraine found that delayed sleep phase disorders were associated with COVID-19, whereas advanced sleep phase disorders, irregular sleep phase disorders, and non-24 h circadian rhythm disorders were not [137].

Long COVID symptoms may either spontaneously resolve or persist, depending on specific neuropsychiatric symptoms. While there is no established treatment specifically for long COVID, various psychological and pharmacological interventions have been explored. These include rehabilitation programs for fatigue and cognition, psychological interventions for anxiety, and cognitive processing therapy for PTSD. Most of these treatments have reported significant improvements, but these results should be considered preliminary [137].

In Table 3, the main selected studies on psychiatric symptoms in adult patients with long COVID are summarized.

#### 5.2.4. Pulmonary Function and Imaging 

SARS-CoV-2 leads to acute viral respiratory tract infections, including pneumonia. Following the initial infection with SARS-CoV-2 in the upper respiratory tract, viral replication persists in the lower airways and alveolar epithelial cells. This triggers a hyper-inflammatory immune response, causing damage to the alveoli and vascular leakage.

Data on the early post-acute COVID-19 phase reveal that up to six months post-infection, COVID-19 patients show a pattern of pulmonary restriction and abnormal carbon monoxide diffusion capacity in lung function testing. Similar results were seen 6–12 months after symptom onset in prospective cohort studies [159,160].

Several studies have examined post-COVID-19 pulmonary function tests (PFTs). A study of 80 patients who underwent both pre-infection and post-infection PFTs showed no difference in forced vital capacity (FVC), forced expiratory volume in 1 s (FEV1), FEV1/FVC ratio, and DLCO. However, total lung capacity (TLC) significantly worsened, correlating with more severe disease (none of the patients were intubated in this study) [161]. Other systematic reviews, comprising seven studies, have indicated that reduced DLCO, restrictive patterns, and obstructive patterns were observed in 39%, 15%, and 7% of patients, respectively, 3 months after COVID-19 infection [162].

Due to severe respiratory inflammation and injury, one of the potential manifestations after COVID-19 is lung fibrosis caused by the cytokine storm [189].

In a follow-up chest CT study of patients after COVID-19, fibrotic-like changes were observed in 35% of the participants. The remaining 65% exhibited either a complete radiologic resolution (38%) or residual ground-glass opacification or interstitial thickening (27%) [163]. Prospective studies assessing these complications will help identify individuals at the highest risk. Advanced imaging studies, combined with physiological markers, may uncover previously unknown or underrecognized pathologies. Overall, persistent ground-glass opacification is the most common radiographic sequela followed by fibrotic changes.

Han et al. investigated the characteristics and time course of pulmonary sequelae following recovery from COVID-19 using regular pulmonary function evaluations and CT scan follow ups. The authors evaluated 67 patients discharged from their hospital with a three-year post-hospitalization follow up. Follow-up visits consisted of anamnesis, physical examinations, PFTs, and HRCTs of the chest. Lung function impairment was measured by performing a pulmonary function test. The recorded parameters included TLC, FVC, residual volume (RV), FEV1, maximum expiratory flow rate (MEF75/25), FEV1/FVC ratio, and DLCO measured by means of the single-breath test. DLCO was expressed as a percentage of predicted normal values and reported as a categorical variable, as defined by the guidelines of the American Thoracic Society (ATS) and European Respiratory Society (ERS). Anomalies were noted as DLCO % predicted with mild reduction (mean value 72% pred) and total lung capacity (TLC) % predicted with mild restrictive lung disease (mean value 72% pred). The mean value for FEV1/FVC was >70 [163].

Computed tomography is a valuable tool for the diagnosis and the follow up of COVID-19 pneumonia, and is considered the reference standard for imaging the chest of patients, providing useful insights into the progression and resolution of the disease [164]. In the early stages of infection, CT scans commonly reveal ground-glass opacities, consolidations, and crazy-paving patterns in the lungs. However, as the patients recover, these findings may gradually lessen, and signs of improvement, such as decreased opacities and resolution of lung involvement, may become evident. In the post-acute phase, CT imaging may still show residual abnormalities, such as fibrotic changes, which can have implications for long-term lung function [165]. Additionally, CT scans can help in identifying potential secondary complications, such as pulmonary embolism or bacterial superinfection, which may arise during the recovery period [166]. 

In a cohort study, 919 patients underwent chest CT scans during a follow-up period. A radiological scoring system was used to quantify the presence of typical parenchymal lung findings associated with COVID-19 pneumonia. The following alterations have been evaluated: ground-glass opacities (GGOs), interstitial thickening, consolidation, bronchiectasis, fibrotic alterations, and honeycombing. Each of the five lung lobes was evaluated individually for each of the described alterations and was assigned a score in the range of 0–5 to indicate the percentage of involvement: 0—absence of lesions, 1—alterations involving <5% of the lobe, 2—lesions involving 5–25% of the lobe, 3—lesions involving 25–50% of the lobe, 4—lesions involving 50–75% of the lobe, and 5—lesions involving >75% of the lobe. The total CT score for each lung was evaluated by summing the scores of the individual lobes. Most of the patients presented fibrotic bands, septal thickening, and bronchiectasis (52, 38, and 23 patients, respectively). Both lungs were involved with multi-lobar affection [166].

It is necessary to follow up COVID-19 patients after their recovery through comprehensive assessments with a focus on respiratory manifestations and, if necessary, begin early treatment.

In Table 3, the main selected studies on pulmonary function and imaging in adult patients with long COVID are summarized.

#### 5.2.5. Long COVID and Cardiovascular Diseases 

The cardiovascular symptoms of long COVID rank as the third most-frequent manifestation of the disease, following neurological and pneumological symptoms. These symptoms result from various cardiac and extracardiac pathological sequelae, including residual respiratory abnormalities, pulmonary hypertension, muscular deconditioning, cytokine dysregulation, left or right ventricular dysfunctions, chronotropic incompetence, altered parasympathetic tone, and increased heart rate variability [190,191,192,193].

Patients who required hospitalization during the acute phase of COVID-19 exhibit more severe cardiovascular symptoms in long COVID compared to those with mild to moderate or asymptomatic cases, and with a significantly higher incidence [167,194,195,196].

Affected individuals may experience hypotonia, palpitation, chest pain, hypertension, elevated blood pressure, tachycardia out of proportion to that expected for effort, and/or drops in oxygen saturation [191]. Several studies have reported cardiac complications and their prevalence in patients with long COVID. A meta-analysis assessed the risk of incident myocarditis within 12 months after COVID-19 recovery: over a mean follow up of 9.5 months, myocarditis occurred in 21/100,000 patients compared with 9/100,000 controls [168].

Another meta-analysis assessed the risk of incident pericarditis within 12 months after COVID-19 recovery. Over a mean follow up of 9.6 months, pericarditis occurred in 3.4/1000 patients compared with 80/100,000 controls [168]. Delayed-onset myocarditis has been described after the viral clearance of SARS-CoV-2, probably triggered by immune-mediated reactions [197], sometimes in the context of the Multisystem Inflammatory Syndrome in Adults (MIS-A) and Children (MIS-C) [198,199], an uncommon SARS-CoV-2 infection complication, characterized by hyperinflammation with cardiovascular involvement [200].

Other cardiac complications and their prevalence in long COVID patients are systolic or diastolic left ventricular dysfunctions (prevalence: 0.06–35%) [169,170,201], coronary artery disease (8%) [173], acute myocardial infarction (1.5–8%) [173], heart failure (0.1–2%) [202], and pulmonary hypertension (10–50%) [203].

Postural Orthostatic Tachycardia Syndrome (POTS) is also described in patients with long COVID [204,205,206]. Patients with POTS typically experience a heart rate increase of more than 30 beats per minute compared to their resting heart rate after standing quietly for 5–10 min (often exceeding 120 bpm) without orthostatic hypotension. They commonly report postural symptoms, such as palpitations, dizziness, weakness, fatigue, blurred vision, and intolerance to exercise [206]. 

The mechanisms underlying persistent cardiac damage following acute illness are still not well-understood. One potential explanation is a chronic inflammatory response triggered by lingering viral reservoirs in the heart after the initial infection [207]. Another mechanism for delayed damage involves an autoimmune response targeting cardiac antigens due to molecular mimicry [208]. A high-throughput proteome analysis by Wang and colleagues has identified a variety of autoantibodies against humoral and tissue antigens in patients with severe COVID-19 [209,210,211].

Several longitudinal studies focusing on cytokine profiling and proteomics have shown an increased expression of prothrombotics persisting beyond the acute phase of infection [212,213]. This is consistent with growing reports of delayed embolic complications [214,215,216]. Endothelial dysfunction and its associated complications may also arise in patients, with evidence of ongoing impairment observed in younger individuals 3–4 weeks after SARS-CoV-2 infection [217,218].

Different reports have explored the utility of a 12-lead electrocardiogram (ECG) in screening patients for post-acute cardiac manifestations [174,175]. Dynamic ECG changes are common during acute illness, but tend to resolve in the majority of hospitalized patients by 6 months after acute COVID-19 and often resemble those of risk-factor matched controls [171,174,175]. However, sinus arrhythmia remains common in the post-acute phase, presenting as transient or sustained episodes of sinus tachycardia or bradycardia [174,175].

A systematic review of longitudinal observational studies performed on young athletes during post-COVID-19 periods showed that electrocardiographic abnormalities indicative of myocarditis were uncommon [178]. 

ECG combined with the tilt table test is useful for the diagnosis of POTS [219].

Both transthoracic echocardiography and Cardiac Magnetic Resonance (CMR) play crucial roles in diagnosing both acute and chronic cardiac conditions. 

While endomyocardial biopsy serves as the gold standard for histological evaluations in suspected severe cases [176], CMR offers a non-invasive alternative for assessing stable cases. CMR provides valuable insights into various pathological processes, such as myocardial edema, hyperemia, necrosis, and fibrosis, by detecting changes in the tissue’s fundamental magnetic properties [176].

Echocardiography is essential for the early detection of cardiac abnormalities in COVID-19 patients (including suspected myocarditis, Takotsubo syndrome, myocardial infarction, and pericardial effusion), especially when hemodynamic stability is uncertain [220]. Right ventricular dilation and dysfunction are the most commonly observed echocardiographic abnormalities with prognostic implications [172,173,221]. As the acute infection subsides [172], most patients show an improvement in right ventricular abnormalities [173]; left ventricular systolic dysfunction is relatively less common [173].

Myocarditis management and treatment of long COVID syndrome depends on the severity of symptoms, complications, onset, and hemodynamic stability vs. instability. It is recommended that patients experiencing heart failure as a result of COVID-19 myocarditis should be treated by established medical guidelines. This involves the administration of ACE inhibitors, angiotensin receptor blockers (ARBs), angiotensin receptor neprilysin inhibitors (ARNi), vasopressors, β-blockers, and diuretics [222]. Usually, patients with myocarditis and COVID-19 pneumonia who still require supplemental oxygen should receive corticosteroid treatment [223]. The use of intravenous corticosteroids might be considered for those with suspected or confirmed COVID-19 myocarditis accompanied by hemodynamic compromise or MIS-A, a hyperinflammatory state characterized by acute heart failure and cardiogenic shock without sepsis. This approach has been linked to a favorable prognosis in a small series of cases [224]. The empirical use of immunosuppressive therapy, such as corticosteroids, might also be considered for individuals with biopsy-proven severe myocardial inflammatory infiltrates or fulminant myocarditis, taking into account the risk of infection [225].

In regard to pericarditis, the management of this post-COVID-19 manifestation relies on nonsteroidal anti-inflammatory drugs (NSAIDs) that can help alleviate chest pain (e.g., ibuprofen, indomethacin, and aspirin) [226]. Low-dose colchicine or prednisone is administered for persistent chest pain, with a strategy to gradually reduce the dosage, depending on symptoms and clinical assessments [226]. Low doses of steroids are also used to relieve the asthenia that many patients report. Anakinra is less used in these forms of pericarditis. A low-dose beta-blocker or a non-dihydropyridine calcium-channel blocker can be introduced and adjusted gradually to reduce the heart rate. This approach may slightly enhance exercise tolerance, alleviate symptoms, and patients can gradually discontinue these medications as their fitness and activity levels improve. Ivabradine is another option for those experiencing severe fatigue worsened by beta-blockers and calcium-channel blockers.

The duration of the therapy is often long, also due to the numerous relapses that patients experience. The tapering of the therapy should be slow and based on the clinical response and the symptoms.

For patients experiencing PASC, treatment for cardiac manifestations is primarily symptomatic, including the use of anti-vasospastic drugs for those with atypical angina or beta-blockers for palpitations [190]. Treatment strategies for POTS include alpha-1 agonists, steroids, compression garments, and increased fluid and salt intake. Non-steroidal anti-inflammatory drugs may be employed to manage specific symptoms, such as fever and pain [23].

Ultimately, the most effective means of preventing severe complications from COVID-19 is vaccination [227]. The data from an observational study indicate that vaccinations may alleviate long COVID symptoms in 56.7% of cases [177].

In Table 3, the main selected studies on cardiovascular diseases in adult patients with long COVID are summarized.

## 6. Long COVID in Vulnerable Adults 

### 6.1. Long COVID in Pregnant Women 

In the following years, the long-term effects of acute infection have become increasingly important, revealing a wide range of sequelae. Particularly noteworthy is the impact of long COVID on gynecological and obstetric health, emerging as a growing field of interest and study in the medical community.

Compared to the non-obstetric population, pregnant women are considered a population at risk since the incidence of clinical manifestations, severity of symptoms, and associated obstetric morbidity seem to be increased [228].

Different studies have established a higher incidence of obstetric complications (preterm labor, preterm birth, preeclampsia, cesarean delivery, and miscarriage) in patients with moderate and/or severe infections [229,230], but women in general appear to be susceptible to prolonged and diversified effects.

The evidence indicates that long COVID disproportionately affects women, with about twice as many women being impacted compared to men [231]. Additionally, women before menopause are at a higher risk of long COVID, suggesting a potential role of sex hormones in the development of this condition [232]. 

For these reasons, interest in the potential implications of long COVID in gynecology and obstetrics has significantly increased, with particular attention on the impact on menstrual cycles, reproductive health, pregnancy, and maternal well-being. The role of immunological changes and hormonal imbalances is also examined to fully understand how long COVID may influence women’s health in various life stages.

#### 6.1.1. Long COVID and Pregnancy 

The diagnosis and management of long COVID are challenging, more so in pregnant women. Given the overlap with normal pregnancy symptoms, women experiencing fatigue and/or shortness of breath may be at an increased risk of worsening in pregnancy, especially in the third trimester [233].

The impact of COVID-19 infection acquired during pregnancy has been extensively studied and its effects have been demonstrated, such as a significant increase in the risk of preeclampsia [234], elevated maternal and perinatal death rates aligning with each wave of the pandemic [235], and a higher occurrence of neurodevelopmental disorders in infants during the initial 12 months after birth for mothers infected during pregnancy [236]. Although, there is currently no scientifically valid literature that offers insights into the development of the long-term consequences of COVID-19 infection in pregnant women who have recovered from SARS-CoV-2 infection before or during the early weeks of pregnancy. 

Probably, due to the physiological modifications that women experience during the gestation period, the manifestation of long COVID symptoms may differ from what has been observed in the general population or in non-pregnant women. 

Long COVID in pregnancy represents a multisystem disorder with significant diagnostic challenges, as more than 200 symptoms associated with the disorder can easily be mistaken for other common conditions.

Limited research has delved into the impact of long COVID on pregnant individuals. An Ecuadorian cross-sectional survey indicated that pregnant (*n* = 16) and non-pregnant (*n* = 231) women with long COVID exhibited similar symptoms, with fatigue, hair loss, and difficulty concentrating being the top-three reported symptoms for both groups [237].

Also, a single-center, cross-sectional, retrospective study on 99 pregnant women who were polymerase chain reaction (PCR)-positive for COVID-19, conducted by Kandemir, showed that many women experienced long COVID after suffering acute COVID-19 during pregnancy, but long COVID prevalence was similar to the general population. In particular, long COVID correlates with the severity, type, and number of symptoms of acute COVID-19 [238]. 

A prospective cohort study involving a longitudinal cohort of ambulatory pregnant patients in the United States revealed that 25% experienced long COVID symptoms eight or more weeks after testing positive for SARS-CoV-2 [239]. 

#### 6.1.2. Post-Viral Fatigue in Pregnant Women

A longitudinal comparative study assessed post-viral fatigue (PVF) following SARS-CoV-2 infection during pregnancy, revealing a higher prevalence of PVF in women infected during pregnancy. The risk of developing fatigue and its duration increased with the severity of the infection [240].

However, investigations into how long COVID specifically affects pregnancy are still lacking.

A significant but relatively small control-matched prospective cohort study conducted in Brazil monitored pregnant women following a positive COVID-19 diagnosis (*n* = 84), revealing that 75.9% developed long COVID. This study also highlighted that pregnant individuals administered glucocorticoids for treating COVID-19 were at a higher risk (RR 6.92, 95% CI 1.70–28.07) of persistent fatigue, a pivotal and debilitating symptom associated with long COVID [241]. 

A more recent study involved 409 pregnant women diagnosed with acute COVID-19, with 286 participants followed up for an average of 92 weeks. Among these women, 140 displayed post-COVID-19 symptoms three months after infection, with neurological (60%) and cutaneous (55%) manifestations being prevalent. The following profiles were identified as having an elevated risk of developing post-COVID-19 conditions: migrant women born in countries with a lower human development index; multiparous women; women who contracted COVID-19 during pregnancy, with a higher number of symptoms and experiencing a greater incidence of moderate and severe symptoms; and women who required hospitalization due to COVID-19 complications and who were not vaccinated prior to the onset of the disease. Perinatal outcomes showed no significant differences.

Women infected during successive pandemic waves consistently exhibited a decrease in the risk of post-COVID-19 conditions. Certain symptoms, especially myalgia and arthralgia, persisted for an extended period before resolving, and a small but notable proportion experienced chronic neurological and psycho-emotional symptoms after 90 weeks. In total, 34.2% of obstetric patients with acute COVID-19 displayed symptoms of post-COVID-19 conditions, highlighting demographic and disease-related risk factors [242].

#### 6.1.3. Prevention of the Post-COVID-19 Condition in Pregnant Women

One of the potential future consequences of long COVID symptoms in pregnant women could involve a rise in the frequency of prenatal medical visits, primarily due to the aftermath experienced by pregnant patients, as previously observed in the Swiss population [243]. 

This not only places a substantial burden on healthcare systems, but also heightens the likelihood of pregnant women contracting hospital-associated illnesses, including infections like COVID-19. 

Consequently, the ongoing implementation of protective measures remains crucial for pregnant women grappling with long COVID. These measures, including mask usage, social distancing, and vaccination, stand out as cost-effective and efficient strategies. 

Furthermore, the obstetric care for pregnant women dealing with long COVID sequelae should be personalized to ensure the well-being of both the mother and the fetus [244]. 

The summary of main studies on long COVID in pregnant women is reported in Table 4.

### 6.2. Long COVID in Cancer Patients

The impact of long COVID on cancer patients adds another layer of complexity. Cancer patients, who are often immunocompromised due to their disease and/or treatment, may face unique challenges when dealing with long COVID. The interplay between the immune system, the effects of cancer treatments, and the persistent inflammatory response associated with long COVID can complicate the recovery process for these individuals. Research has indicated that cancer survivors, particularly those who have undergone treatments like chemotherapy or stem cell transplantation, may be at an increased risk of experiencing prolonged symptoms of fatigue, respiratory issues, cognitive difficulties, and other lingering effects associated with long COVID. The pre-existing vulnerabilities in these patients’ immune systems may contribute to a protracted recovery period.

#### 6.2.1. Epidemiological Data for Cancer Patients

The data available for cancer patients and long COVID are heterogeneous, mainly due to the different populations examined and the period of observation. 

Most data, represented in three of the nine studies analyzed, were collected from the OnCovid European Registry study.

Pinato et al. conducted their analysis on an active European OnCovid registry that involved 37 institutions across six countries [245]. The data lock for the analysis was 1 March 2021, and only 7% of the patients had at least one dose of the anti-SARS-CoV-2 vaccine. The prevalence of long COVID was 15%. The most common sequelae included respiratory symptoms (49.6%), residual fatigue (41%), weight loss (5.5%), neurocognitive symptoms (7.3%), non-respiratory organ dysfunction (1.7%), and other complications (18.4%). The highest prevalence of sequelae was observed in men compared to women (*p* = 0.041), aged 65 years or older, compared with other age groups (*p* = 0.048); those with two or more comorbidities compared with one or none (*p* = 0.0006) had a history of smoking compared with no history (*p* = 0.0004); those with higher rates of COVID-19 complications (*p* < 0.0001), requiring therapy for COVID-19 (*p* = 0.0002); and requiring hospitalization (*p* < 0.0001). The distribution of primary tumors was also significant different between the two groups (*p* = 0.048) [246].

An updated follow up of the OnCovid registry was conducted by Cortellini et al., with a database lock of 2 February 2022 [247]. The prevalence of sequelae from COVID-19 was 16.6%, with 9% having respiratory sequelae, 7% prolonged fatigue, 1.6% weight loss, 2.9% neurocognitive sequelae, and 1.3% reporting other categories of organ dysfunction. The authors conducted several analyses to evaluate the impact of vaccinations, the Alpha–Delta phase, and the Omicron phase on the onset of long COVID sequelae and risk factors. Unvaccinated patients were at a high risk of experiencing COVID-19 sequelae regardless of the viral strain. When comparing unvaccinated and vaccinated patients, the prevalence of post-COVID-19 sequelae was higher among current or former smokers. Patients who received a booster dose and those who received two vaccine doses had a significantly lower prevalence of overall COVID-19 sequelae compared to unvaccinated patients.

Thereafter, a long-term follow up on the OnCovid registry was performed with the aim to describe the prevalence and type of COVID-19 sequelae at 6 and 12 months [248]. The prevalence was 9.8% and 8% at 6 and 12 months, respectively. At the 6-month follow up, 4.9% had respiratory sequelae, 3.2% fatigue, 2.2% neurocognitive sequelae, and 2.7% other kinds. At the 12-month follow up, 3% had respiratory sequelae, 3% had fatigue, 1% had neurocognitive sequelae, and 3% had other types. Patients suffering from sequelae were more likely smokers (*p* = 0.007) and had a history of complicated COVID-19 (*p* = 0.0223).

An Italian prospective study on COVID-19 cancer patients collected data on the incidence of long COVID symptoms by a telephonic survey [249]. Twelve patients (12.4%) reported sequelae, all who were vaccinated with three doses of mRNA vaccines. Specifically, 33.3% reported myalgia, while the majority reported fatigue (58.3%) and brain fog (50%). Only 25% had respiratory symptoms. In the univariate analyses, only the female sex (*p* = 0.024), obesity (*p* = 0.039), and diabetes mellitus (*p* = 0.014) were shown to relate to the occurrence of long COVID symptoms.

One-hundred cancer patients receiving care at the University of Texas MD Anderson Cancer Center were screened by questionnaires for 14 days after COVID-19 infection, then weekly for three months, and then monthly thereafter [250]. More than half reported symptoms persisting at least 30 days after the COVID-19 diagnosis. The most commonly reported symptoms were fatigue (82%), sleep disturbance (78%), myalgia (67%), gastrointestinal symptoms (62%), headache (47%), altered smell and taste (47%), dyspnea (47%), and cough (46%). It is noteworthy that none of these patients were vaccinated. 

Another monocenter study involved oncological patients at Guy’s Cancer Center in London [251]. Patients were contacted by using telephone survey. Symptoms were described according NICE guidelines [185] and classified as long COVID if they persisted over 4 weeks after COVID-19 diagnosis. Half of the patients investigated developed long COVID. The most frequently reported symptoms by patients included fatigue (78%), breathlessness (53.7%), cognitive impairment (46.3%), sleep disturbances (39%), loss of taste (34.1%), and depression (29.3%). Breast, lung, and CNS cancers were the cancer types most commonly associated with long COVID. There were no significant differences observed between the disease stage and the incidence of long COVID.

A retrospective international multicenter study was conducted with the aim to describe the outcomes of hematological patients infected by SARS-CoV-2, and the authors reported data on long COVID sequelae [252]. The incidence of post-COVID-19 symptoms at 3 and 6 months was 7.5% and 9.2%, respectively. 

A multicenter retrospective study conducted through a combination of in-person visits, EMR data, and telephone calls documented an incidence of long COVID symptoms in 30% of cancer patients [253]. The incidence at 12 months was lower than 6 months (8.3 vs. 15%). The symptoms were classified as respiratory (cough, shortness of breath, dyspnea, wheezing, hypoxia, etc.), constitutional (fatigue, weakness, poor appetite, weight loss, etc.), psychiatric (delirium, brain fog, insomnia, anxiety, etc.), and neurologic (memory deficit, dysgeusia, anosmia, headache, etc.). The most common sequelae at 6 and 12 months were respiratory (2.4% vs. 2.8%), constitutional (12% vs. 2.8%), and psychiatric (2.4% vs. 2.8%).

An original paper was the result of a longitudinal online survey performed during the pandemic in a large cohort of cancer patients [254]. Cohort 2 was designed to investigate symptoms lasting more than 6 months, with 37 patients evaluated, 11 of whom were not vaccinated. The predominant symptoms observed were fatigue at 66%, followed by a loss of taste or smell at 30%, muscle or joint pain at 28%, dizziness at 23%, and respiratory symptoms at 19%. Additionally, more than 10% of the patients reported symptoms like muscle weakness, sleep disturbances, headaches, cough, and palpitations. Those who developed enduring symptoms were predominantly women (although not significantly so) and more commonly had a BMI over 30 or pre-existing conditions, such as hypertension, cardiovascular disease, or asthma.

#### 6.2.2. Risk Factors of Long COVID in Cancer Patients

Numerous risk factors have been associated with the development and severity of COVID-19 infection in cancer patients [89,255]. Hospitalization or treatment for acute infection in cancer patients, as well as a previous history of smoking, male sex, higher comorbidity, and older age, have been considered risks for experiencing PASC, as reported in non-cancer populations [246]. In the only comparative study, no significant difference in the prevalence of long COVID was observed between cancer patients and a non-cancer control cohort in the first 12 months after infection, despite the difference in mortality between the two cohorts [253].

Both hematological and solid cancer patients have a similar incidence of COVID-19 sequelae, although hematological patients have reported the highest rates of anticancer treatment modification and discontinuation [246]. In the population studied by Visentin et al., including patients diagnosed with chronic lymphocytic leukemia (CLL) on active treatment or who received treatment in the previous 12 months, 16% developed post-COVID syndrome, consistent with the frequency observed among patients with cancer in OnCovid studies (15%) [248].

Considering patients without metastatic or advanced disease, as in the study by Cortellini et al., the incidence was 16.6% at the first follow up at 2.3 months from COVID-19 diagnosis, with no association with oncological features or the receipt of anti-cancer treatment within 4 weeks of COVID-19. In smaller series compared with the non-long COVID group, the highest rate of long COVID was observed in breast cancer (17% vs. 5.1), lung cancer (14% vs. 2.6%), and CNS (9.9% vs. 2.6%), without differences in stage [251]. Only breast cancer has been confirmed to be associated with long COVID in Lasagna et al.’s study, where PASC was observed in 12.6% of interviewed patients with solid cancer on treatment [249]. No association between cancer characteristics (e.g., tumor stage or cancer treatment) and COVID-19 sequelae was found in the OnCovid Registry [246]. 

The duration of follow up or the waves analyzed are also important parameters for the prevalence of long COVID. Cortellini et al. showed 9.8% and 8% sequelae present at 6 and 12 months, respectively, after acute infection [248]. He also demonstrated in another study that Omicron infection was associated with a reduction in PASC compared with infections contracted during previous phases of the pandemic (6.2% vs. 16.8%) [247]. This could explain the wide range of PASC described in literature in both non-cancer and cancer patients, respectively, 13–60% and 9.8–60.2% [246,256,257]. 

#### 6.2.3. Vaccines and Long COVID in Cancer Patients

For the first time in the analysis of the OnCovid Registry, in 2022, it was documented that cancer patients with full vaccination were characterized by a lower probability of severe COVID-19 infection and mortality compared to controls. Reduced symptomatic COVID-19, the need for COVID-19-oriented therapy, complications, hospitalizations due to COVID-19, and oxygen therapy requirements were observed. Furthermore, the proportion of patients reporting at least one sequela from COVID-19 was significantly lower in fully vaccinated patients compared to unvaccinated controls (6.7% vs. 17.2%) [245]. More recently, it was confirmed by Cortellini et al., based on the OnCovid Registry with a longer follow-up session, where the data support vaccination as a determinant of the reduction in PASC, when patients received a booster or two vaccine doses [247]. 

A higher incidence of long COVID, with 60.2% of patients reporting symptoms at least 30 days after being diagnosed with COVID-19, has been recorded by Dagher et al. in the unvaccinated population. The authors analyzed cancer patients followed at the MD Anderson Cancer Center during the initial period of the COVID-19 pandemic in March 2020; SARS-CoV-2 vaccines were made available only in late 2020 [250]. In contrast, in CLL-infected patients, there is no protection against PASC with vaccines; nevertheless, vaccinated individuals had a lower hospitalization rate and better overall survival than unvaccinated individuals [252].

#### 6.2.4. Clinical Manifestation of Long COVID in Cancer Patients

While there is certainty about the incidence of PASC in cancer patients, it is more challenging to accurately gauge the extent of the problem and to better characterize its symptoms. As the frequency and presentation of acute COVID-19 signs and symptoms varied between early and late waves, the same observation applies to SARS-CoV-2 sequelae, with inconsistent data across different studies [246,254], likely due to various SARS-CoV-2 variants, patient adherence to shielding measures, and vaccine availability. Similarly, in cancer patients, it can be challenging to differentiate the origin of some symptoms. Asthenia, anorexia, and dyspnea are often symptoms related to the disease itself or to cancer treatments and are unlikely to differ from post-COVID-19 symptoms.

In the studies we analyzed, fatigue, headaches, muscle or joint pains, and respiratory and neurocognitive sequelae were the most prevalent symptoms described, with a wide range of variability. In Cortellini et al.’s study, a non-metastatic/advanced cohort of patients showed at least one COVID-19 sequela in 16.6% of patients, including fatigue, neurocognitive, and respiratory sequelae in 7.1%, 3.8%, and 7.6%, respectively [248]. 

In a smaller population, Lasagna et al. reported fatigue as the main symptom (58%), followed by brain fog (50%), myalgia (33.3%), and respiratory symptoms (25%) [249]. Fatigue is the symptom most commonly reported as persistent over the long-term follow up.

#### 6.2.5. Long COVID Syndrome and Cancer Outcome

It has been observed that patients experiencing any sequelae from COVID-19 infection face an increased risk of death because these sequelae can lead to the deferral of oncological care. 

For patients undergoing cancer treatment at the time of COVID-19 diagnosis, it has been demonstrated that those who resumed treatment with systemic anticancer therapy after regimen adjustments had similar post-COVID-19 survival rates to those who resumed or continued their treatment without changes. However, those who permanently discontinued treatment experienced a significantly increased risk of death [246]. It is evident how the persistence of one or more symptoms related to COVID-19 infection can significantly influence the decision and ability to resume cancer treatment. Therefore, it is essential to provide information and training to healthcare workers to properly recognize long COVID and collaborate with multidisciplinary teams to offer appropriate medical and rehabilitation support, thereby improving the patient’s quality of life and promoting the prompt resumption of cancer programs.

The symptoms reported by patients with long COVID often overlap considerably with those experienced by cancer survivors and those occurring during chemotherapy, radiotherapy, targeted therapy, and immunotherapy [11,258]. NICE defines the long-term effects of COVID-19 syndrome as symptoms that persist or develop after acute COVID-19 infection and cannot be explained by an alternative diagnosis. Therefore, if we analyze the WHO’s definition of long COVID, which defines sequelae that cannot be attributed to other causes, it raises the question: “does it also apply to the oncology patient?”.

The summary of the main studies on long COVID in cancer patients is presented in Table 5.

## 7. Integrated Approach to Long COVID Management

An integrated approach is crucial for long COVID management. A multidisciplinary team is mandatory because of the multiorgan involvement typical of this condition [15,34]. 

Fainardi et al. proposed a three-step approach that includes a screening phase, an assessment phase, and, lastly, a monitoring phase. Firstly, according to NICE recommendations, a questionnaire could be administered to determine the acute phase of the disease and assess the symptoms of long COVID [185], but currently a specific questionnaire for children is not available. 

We proposed a multidisciplinary questionnaire for both adults (Supplementary Materials Document S1) and children (Supplementary Materials Document S2) with the aim of facilitating early recognition and appropriate management. The questionnaires, meant to be administered by medical personnel, are based on our previous experience of COVID-19 follow-up sessions at 1 year after infection [259] and the scientific evidence presented above. The questionnaires investigate the presence of the main symptoms linked to COVID-19 sequelae and related to pulmonary, neurological, and gastrointestinal symptoms, also characterizing patients for previous diseases and conditions, the severity and treatment received during the acute infection, the overall number of infections, and the vaccination status at infection. These tools will not only be valuable to identify long COVID patients, but also highlight the relationship between long COVID syndrome prevalence and the factors related to acute infection, and hopefully the protective effect of vaccinations.

A physical examination is essential to detect clinical signs, and then appropriate diagnostic tests should be chosen. 

Nutritional management also has a great relevance. Weight loss or gain should be carefully monitored, and referrals to dietetics should be made as necessary. Emphasis should be placed on maintaining a well-balanced diet without excluding any food groups unless medically indicated. Meals may need to be smaller and more frequent to better manage dietary needs. Likewise, the qualitative aspects of the diet and the dietary pattern pursued should be monitored in patients affected by COVID-19. In fact, the consumption of foods high in saturated fats, sugars, and refined carbohydrates (some typical features of the Western diet (WD)) contribute to the prevalence of obesity and type 2 diabetes, and could place individuals at an increased risk of severe COVID-19 pathology and mortality. WD consumption triggers the innate immune system and inhibits adaptive immunity, resulting in persistent inflammation and reduced host protection against viruses. To prevent long-term consequences from COVID-19, individuals should prioritize healthy eating habits, consuming high amounts of fiber, whole grains, unsaturated fats, and antioxidants to boost immune function [260]. Dietetic support may be necessary to ensure adequate calorie intake and meet nutritional requirements. The observed changes in body composition were also driven by the changes in physical activity habits. 

Therefore, the establishment of sedentary habits and COVID-19-related long-term symptoms may have contributed to the excessive accumulation of fat mass while detrimentally affecting lean mass. A sedentary lifestyle is counterproductive, as proper and tailored exercises stand as a promising, effective therapy for mitigating post-COVID-19 symptoms and helping people in recovering faster and increasing their autonomy, functionality, and quality of life [261]. Thus, to guide the recovery, it may be useful to identify an early resumption of unstructured physical activity (i.e., walking, cycling, dancing, playing at the park, and performing housework) alongside targeted nutritional intervention as a useful therapeutical approach to prevent poor nutritional and sedentary habits resulting in excessive fat mass accumulation. Exercise prescription should be approached with caution in patients experiencing CFS and post-exertional malaise, as exercise is either not recommended or considered unhelpful in terms of effectiveness and safety, respectively [262].

Well-being and psychological support are essential components of care and should be tailored to each individual patient. 

There is no single diagnostic test for long COVID. Several non-invasive diagnostic tests could be helpful, such as blood exams, ECG and echocardiography, neurologic evaluations, polysomnography, sniffing test, audiometry, abdominal ultrasound, 6MWT, lung functioning tests (e.g., spirometry), and LUS [15].

The last phase could be performed as a regular follow up of lung functioning at 3, 6, and 12 months and a rehabilitation program with light aerobic exercise according to patients’s abilities. 

While several guidelines regarding the management of long COVID have been issued, there still exists a significant practical gap, and specific treatments have not been thoroughly reviewed. There is no current effective pharmaceutical treatment for long COVID syndrome [23]. Medical treatment with paracetamol and non-steroidal anti-inflammatory drugs, bronchodilators, or inhaled steroids could be used to manage specific symptoms. 

In patients with persistent and severe mental symptoms, psychological interventions with psychological support are mandatory [55]. Pediatric patients should be encouraged to return to school. Providing letters for school to allow for a flexible reduced timetable can often make school attendance more manageable on a consistent basis. Once a manageable baseline timetable has been established, it should be maintained for several weeks before gradually increasing.

Children with an increased risk of long COVID and their parents or guardians should be informed about the possibility of developing this condition after the acute infection so that early interventions can be performed. Long COVID indeed has an important impact on patients’ quality of life.

Based on our experience [263], telemedicine proves to be a valuable tool for the management of long COVID. Telemedicine allows for the remote monitoring of vital signs like oxygen saturation, heart rate, and respiratory rate. This can help healthcare professionals identify any potential complications early on and prevent the worsening of the condition. Doctors can conduct consultations remotely, assess symptoms, and recommend treatment plans for long COVID patients. This can include medications, physical therapy recommendations, or mental health support. 

The prognosis for many adults, children, and young people is favorable. Many show improvements within 6 months of diagnosis, with a notable enhancement between 3 and 6 months.

Currently, there are no effective strategies to prevent the development of long COVID after acute infection. The only available strategy is to avoid SARS-CoV-2 infection through vaccination [264].

## 8. Limitations

Our work certainly has limitations. Firstly, we present a narrative review that, as noted by Gregory et al. [265], provides a non-systematic overview and analysis of the existing literature on a specific topic. Due to its non-systematic approach, there are no formally established guidelines for conducting narrative reviews, which can introduce potential biases in selection and often results in qualitative syntheses. Consequently, our review methodology possesses inherent limitations. Specifically, during the search, all established definitions of long COVID were included. By employing more specific and unique definitions, it might have been possible to identify articles that meet similar criteria and can articulate more nuanced conclusions. 

Secondly, the primary aim of our review was to provide a comprehensive overview of the incidence, clinical characteristics, risk factors, and outcomes of persistent COVID-19 symptoms in children, adults, and vulnerable populations, rather than focusing solely on pathogenetic mechanisms. Consequently, we included a section on pathogenetic mechanisms without conducting a dedicated review on this topic. A more detailed examination of a subset of articles focusing on pathogenic mechanisms could have facilitated the analytical categorization of associated characteristics, such as the presence or absence of organ damage requiring treatment, and allowed for comparative analyses.

Finally, we introduce the context of chronic diseases, considering the bidirectional relationship with COVID-19, viewing them more as risk factors. However, dedicated studies on patients with chronic diseases could be useful in identifying how symptoms may affect and interfere with these conditions.

## 9. Conclusions 

The outbreak of the pandemic caused by the SARS-CoV-2 virus has necessitated an incredible effort from medical and scientific resources to address the severe symptoms induced by acute infection, even leading to death.

In the following years, the long-term effects of acute infection have become increasingly important, revealing a wide range of sequelae, both in children and adults. Long COVID is a complex multisystem disorder believed to result from a dysregulated immunological response. The incidence and severity of long COVID symptoms can have a significant impact on the quality of life of patients and the course of disease in the case of pre-existing pathologies. Particularly, in vulnerable patients, the presence of PASC is related to significantly worse survival independent from pre-existing vulnerabilities and treatment; it is important try to achieve early recognition and management. Various mechanisms are implicated, resulting in a wide range of clinical presentations.

Understanding the specific mechanisms and risk factors involved in long COVID is crucial for tailoring effective interventions and support strategies. Management approaches involve comprehensive biopsychosocial assessments and the treatment of symptoms and comorbidities, such as autonomic dysfunction, as well as multidisciplinary rehabilitation. This approach, coupled with attention to symptom management and activity pacing to achieve functional goals, along with a supported return to education, has proven effective for most patients. The overall course of long COVID is one of gradual improvement, with recovery observed in the majority, though not all, of patients. Specifically, severe fatigue or post-exertional malaise are symptoms that can persist, both in adults and children [266]. Post-viral fatigue is a common feature observed in both post-COVID-19 and post-EBV conditions. A deeper understanding of post-COVID-19 as a distinct condition, while acknowledging its similarities with other post-viral syndromes, is essential. The healthcare burden and socio-economic implications for patients and their families necessitate additional research and the formulation of suitable healthcare management strategies.

As research on long COVID continues to evolve, ongoing studies are likely to shed more light on the intricate relationship between chronic diseases, such as oncological status, cardiovascular diseases, psychiatric disorders, and the persistent effects of SARS-CoV-2 infection. This information could guide healthcare providers, researchers, and policymakers in developing targeted interventions.

## Figures and Tables

**Table 1:** List of keywords used in our literature search divided by age and system or organ and respective type and number of articles found.

Population/Organs	Keywords	Number of Potentially Relevant Manuscripts	Number of Considered Manuscripts	Number and Type of Articles
Pediatric populations				
Pediatrics	Long COVID or Post-COVID-19 syndrome or Post-Acute Sequelae of COVID-19 syndrome AND Children Or Adolescents or Pediatric age	98	60	23 reviews 21 cohort studies 7 cross-sectional studies 2 retrospective studies 4 meta-analyses 3 clinical practice guidelines
Adult populations				
Neurological and Psychiatry symptoms	Long COVID and/or post-COVID-19 condition and psychiatric symptoms	366	35	5 systematic reviews and meta-analyses 22 cohort studies and longitudinal studies 3 narrative reviews 5 cross-sectional studies
Dermatological manifestations	Long COVID-19 and skin; long COVID and skin; long COVID syndrome and skin; cutaneous skin COVID-19; cutaneous long COVID	50	12	1 systematic review and meta-analysis 4 narrative reviews 7 cohort studies/experimental research
Gastrointestinal symptoms	Gastrointestinal symptoms of long COVID; long-haul COVID-19 gastrointestinal problems; digestive issues after COVID-19 infection; post-COVID-19 gut health; persistent gastrointestinal symptoms following SARS-CoV-2; COVID-19 and chronic digestive disorders; long COVID and irritable bowel syndrome	86	12	2 narrative reviews 1 systemaic review and meta-analysis 7 cohort studies 2 case–control studies
Lung function	Lung involvement in long COVID; CT findings for long COVID; lung function tests in long COVID	32	9	6 restrospective/follow-up, longitudinal studies 2 reviews 1 cross-sectional study
Cardiovascular manifestations	Long COVID; myocarditis; cardiovascular outcome for long COVID; cardiovascular sequelae in long COVID	40	14	2 narrative reviews 7 cohort studies 1 meta-analysis 3 observational studies 1 consensus expert opinion
Vulnerable populations				
Pregnant women	COVID-19; SARS-CoV-2; pregnancy; long COVID and pregnancy; long COVID and obstetric complications; Sequalae; women	90	16	4 systematic reviews and meta-analyses 3 prospective multicenter cohort studies 2 editorials 1 retrospective cohort study 1 cross-sectional retrospective study 2 prospective cohort studies 1 observational prospective study 1 population-based cohort study 1 longitudinal comparative study
Cancer patients	Long COVID AND cancer patients; long COVID AND cancer	60	9	7 retrospective cohort studies 2 ambidirectional cohort studies

**Table 2:** Main studies on long COVID in pediatric-age patients.

First Author; Year of Publication	Study Type	Population	Results
Ashkenazi-Hoffnung et al., 2021 [39]	Prospective cohort study	90	60% symptoms associated with functional impairment
Kikkenborg et al., 2022 [41]	Cross-sectional study	24,315	61.9% long COVID symptoms
Bloise et al., 2022 [35]	Meta-analysis	1413	20% long-term symptoms
Borch et al., 2022 [40]	Cohort study	37,522	Most common symptom is fatigue
Buonsenso et al., 2022 [70]	Cohort study	510	87.4% persistent symptoms
Dennis et al., 2021 [34]	Prospective study	201	60% severe post-COVID-19 syndrome
Parisi et al., 2021 [66]	Cross-sectional survey	267	20% persistent symptoms
Miller et al., 2022 [36]	Cohort study	5032	2.6% persistent symptoms
Molteni et al., 2021 [73]	Prospective cohort study	1734	4.4% long-lasting symptoms
Stephenson et al., 2023 [28]	Prospective cohort study	3395	66.5% symptoms after 3 months
Calcaterra et al., 2024 [37]	Prospective cohort study	167	16.5% reported experiencing at least one symptom indicative of long COVID

**Table 3:** Main selected studies on multisystemic symptoms in adult patients with long COVID.

First Author; Year of Publication	Study Type	Population	Results
Cutaneous manifestations			
McMahon et al., 2021 [108]	Multicenter registry study	Confirmed or suspected COVID-19 patients with dermatological manifestations	One patient exibited a chilblain-like acral and livedo reticularis pattern for over 22 weeks
Tammaro et al., 2021 [110]	Observational study (cohort study)	Patients with persisting cutaneous lesions that appeared during the acute peak of COVID-19	Confluent erythematous/maculopapular/morbilliform rash and papulovesicular exanthem lasted for more than 26 weeks after the acute peak of COVID-19
Förster et al., 2022 [111]	Observational study (cohort study)	Patients with a confirmed diagnosis of COVID-19 (both hospitalized and non-hospitalized)	Long-term (more than 12 weeks) cutaneous sequelae in 15 out of 127 hospitalized patients and in 26 out of 588 non-hospitalized subjects
Huang et al., 2021 [112]	Bidirectional cohort study	Patients with confirmed COVID-19 and discharged from hospital	3% of patients reported skin rashes 26 weeks after disease onset
Garrigues et al., 2020 [113]	Cohort study	Discharged COVID-19 patients	Hair loss in 20% of patients, 110 days after hospital discharge
Grieco et al., 2022 [114]	Experimental study	COVID-19 and long COVID patients	Hypertrophy of dermal nerve fibers in all long COVID study patients. In 2 long COVID subjects, we noted an alteration in dermal A delta and C neuronal fibers
Hussain et al., 2024 [105]	Experimental study	Murine Hepatitis Virus-1 (MHV-1)	Absence of hair follicles, destruction of adipose tissues, and obliteration of the epidermal layer as long-term skin effects. SPIKENET peptide able to restore long-term cutaneous changes
Gastrointestinal manifestations			
Bogariu et al., 2022 [116]	Narrative review	-	The SARS-CoV-2 virus can affect any part of the digestive system
Choudhury et al., 2022 [117]	Systematic review and meta-analysis	50 studies included	GI symptoms were seen in 12% of patients after COVID-19 and 22% as a result of long COVID. Loss of appetite, dyspepsia, IBS, loss of taste, and abdominal pain were the 5 most common GI symptoms of long COVID
Marasco et al., 2023 [118]	Case–control study	883 hospitalized patients (614 patients with COVID-19 and 269 controls)	Compared with the controls, hospitalized patients with COVID-19 had fewer problems of constipation and hard stools at 12 months after acute infection. Patients with COVID-19 had significantly higher rates of IBS than controls
Golla et al., 2023 [119]	Case–control study	320 patients with COVID-19 and 2 control groups: 320 healthy spouses/family controls and 280 healthy COVID-19 serology-negative controls	COVID-19 led to a significantly higher number of new onset PI-FGID/DGBIs compared with healthy controls at 3 and 6 months of follow-up
Fernandez-de-las-Penas et al., 2023 [120]	Cohort study	1266 previously hospitalized COVID-19 survivors	The prevalence of gastrointestinal post-COVID-19 symptoms fluctuates over the first few years after infection, showing a progressive decrease
Zhang et al., 2023 [121]	Cohort study	983 antibiotic-naïve patients with mild COVID-19	3-month samples collected from patients with GI symptoms associated with long COVID showed significant differences, and the ectopic colonization of the oral cavity by gut microbes was observed at the 3-month timepoint
Zuo et al., 2023 [122]	Cohort study	Two cohorts of healthy, young, Chinese subjects (*n* = 54 and *n* = 62)	Baseline gut microbiome composition was intricately associated with different COVID-19 manifestations, particularly GI involvement and post-COVID-19 lingering symptoms
Plummer et al., 2023 [123]	Narrative review	-	Neurocognitive PACS symptoms may endure because viral damage to the blood–brain and intestinal barriers could permit an unchecked flow of harmful substances produced in the gut lumen in the context of dysbiosis
Elmunzer et al., 2023 [124]	Cohort study	5116 patients	Extended GI manifestations were linked to the severity of GI symptoms and the extent of psychological trauma associated with the illness experience
Yagi et al., 2023 [125]	Cohort study	943 COVID-19 patients	6.2% of patients had GI long COVID symptoms; the health-related QoL parameters in patients with GI long COVID symptoms were significantly lower than in those without GI long COVID symptoms, with more varied long COVID symptoms compared to patients without GI symptoms
Ashktorab et al., 2023 [126]	Cohort study	747 patients with positive SARS-CoV-2 PCR	Dyspeptic symptoms were common GI manifestations in the acute and post-COVID-19 periods. GI symptoms, abnormal LFTs, and a history of liver disease during the acute infectious phase associated with abnormal MoCA and sleep problems during follow-up
Ma et al., 2024 [127]	Retrospective cohort study	COVID-19 group (*n* = 112,311) Contemporary (*n* = 359,671) and historical comparison groups (*n* = 370,979)	The risks of developing digestive diseases showed a stepwise increase with the severity of COVID-19
Neurological and psychiatric manifestations			
Akbarialiabad et al., 2021 [128]	Systematic review	120 publications included	Major mood swings, depression, feelings of loneliness and isolation, stress and anxiety, and sleep–wake disorders
Alemanno et al., 2021 [129]	Observational study	87 patients	Patients with severe functional impairments were significantly influenced by cognitive and emotional deficits
Buoite Stella et al., 2022 [130]	Prospective observational study	Participants recruited among the post-COVID-19 ambulatory services	Orthostatic intolerance; sudomotor, gastrointestinal, and pupillomotor abnormalities; decreased tolerance to environmental conditions; and sexual impairments were commonly reported
Chamberlain et al., 2021 [131]	Observational study	Survivors of suspected or confirmed COVID-19 recruited from the general population, predominantly in UK	PTSD symptoms disproportionately elevated in severe forms of COVID-19 (inpatient admission and ventilation support), compared with mild COVID-19 forms managed at home
Chaumont et al., 2022 [132]	Multicenter observational study	Multicenter registry of 222 adult patients	Neuro-COVID-19 carries a high risk of long-term neuropsychiatric disabilities, e.g., impaired cognition, persistent smell/taste disorders, memory complaints, anxiety, or depression
Del Brutto et al., 2021 [133]	Longitudinal prospective study	Stroke- and seizure-free individuals with pre-pandemic cognitive assessments, normal brain MRI, and EEG recordings	Cognitive decline among individuals with mild symptomatic SARS-CoV-2 infections
Del Brutto et al., 2022 [134]	Longitudinal prospective study	COVID-19 survivors and noninfected individuals	Cognitive decline associated with long COVID may naturally diminish over time
Fernández-de-las-Peñas et al., 2022 [46]	Multicenter cohort study	1969 individuals	Female sex was a risk factor for the development of some long-term post-COVID-19 symptoms, including mood disorders, e.g., fatigue, pain, depressive levels, and worse sleep quality
Frontera et al., 2021 [135]	Prospective observational study	382 hospitalized COVID-19 patients who survived and had new neurological issues vs. control group (395 hospitalized COVID-19 survivors without neurological problems)	Abnormalities in functional outcomes, ADLs, fatigue, abnormal cognition, anxiety, depression, and sleep occurred in >90% of patients 6 months after hospitalization for COVID-19
Graham et al., 2021 [136]	Prospective study	50 SARS-CoV-2 laboratory-positive patients 50 laboratory-negative with suspected COVID-19	Non-hospitalized long COVID patients experience mainly brain fog and fatigue that affect their cognition and QoL; among the other symptoms are headaches; numbness/tingling; dysgeusia; anosmia; myalgia; depression/anxiety; dizziness; pain; insomnia; short-term memory deficit; blurred vision; tinnitus; and attention deficit
Huang et al., 2021 [112]	Cohort study	1733 patients	Fatigue or muscle weakness, sleep disorders, anxiety, or depression were commonly reported
Kubota et al., 2023 [137]	Narrative review	-	Fatigue, cognitive impairment, sleep disorders, depression, anxiety, and PTSD
Lauria et al., 2022 [138]	Single-center study	100 elderly individuals assessed on average 3 months after acute COVID-19	COVID-19 elicits persistent measurable neurocognitive alterations in the elderly, particularly in the areas of attention and working memory
Magnúsdóttir et al., 2022 [139]	Prospective cohort study	Seven cohorts across six countries (Denmark, Estonia, Iceland, Norway, Sweden, and the UK) aged 18 years or older, up to 16 months after a diagnosis of COVID-19	Individuals diagnosed with COVID-19 showed a higher prevalence of depressive symptoms and reduced sleep quality, but did not differ in terms of anxiety symptoms or COVID-19-related distress, compared to those without a COVID-19 diagnosis
Marchi et al., 2023 [140]	Systematic review	33 reports: 282,711 long COVID patients	Symptoms mostly associated with Long COVID were depression, anxiety, cognitive and sleep disturbances, and PTSD
Martino et al., 2022 [141]	Prospective monocentric cohort study	64 total patients	Reports after 6 months: anxiety (48.5%), depression (56.4%), persistent fatigue (37.5%), and memory and attention deficits (11%) Reports after 12 months: anxiety (50%), depression (61%), persistent fatigue (12.5%), and memory and attention deficits (4.7%)
Mattioli et al., 2021 [142]	Prospective monocentric observational study	120 healthcare workers previously affected by COVID-19	No evidence of neurological deficits or cognitive impairments in this specific group of patients with mild to moderate COVID-19 4 months after diagnosis. Severe emotional disturbances in those who had COVID-19 confirmed
Mazza et al., 2021 [143]	Prospective cohort study	226 COVID-19 survivors	The following were observed in the sample: persistent depressive symptomatology, poor performance in at least one cognitive domain (78%), with impaired executive functions and psychomotor coordination in 50% and 57% of the sample PTSD, anxiety, and insomnia decreased during follow-up session
Mazza et al., 2020 [144]	Cohort study	402 patients surviving COVID-19	COVID-19 survivors presented a high prevalence of emergent psychiatric sequelae (55% of the sample had a pathological score for at least one disorder) higher than the average incidence of PTSD, major depression, and anxiety
Naik et al., 2021 [145]	Prospective observational study	1234 patients: median duration of follow-up session at 91 days	The most common neuro-long COVID symptoms included fatigue, insomnia, mood disturbances, and anxiety
Nalbandian et al., 2021 [11]	Narrative review	-	Psychiatric symptoms: anxiety, depression, sleep disturbances, and PTSD
Natarajan et al., 2023 [146]	Systematic review and meta-analysis	36 studies: 11,598 long COVID patients	Mental health symptoms: depression, anxiety, PTSD, and sleep disturbances
Premraj et al., 2022 [147]	Meta-analysis	18 studies: 10,530 patients	Psychiatric manifestations (sleep disturbances, anxiety, and depression) are common and increase significantly in prevalence over time
Pistarini et al., 2021 [148]	Cross-sectional and exploratory study	40 patients admitted to rehabilitation units	Patients who recovered in COVID-19 and post-COVID-19 functional rehabilitation units presented with cognitive deficits
Poletti et al., 2022 [149]	Observational study	312 COVID-19 survivors vs. control group	Neuropsychiatric consequences of COVID-19 persistent after hospital discharge (partial improvement at 6 months). Cognitive impairment, in association with depression, has a detrimental impact on QoL
Schou et al., 2021 [150]	Systematic review	66 studies: 266,586 participants	Depression/anxiety, PTSD, cognition, fatigue, sleep disturbances, and psychotic disorders
Seeβle et al., 2021 [151]	Single-center study	96 patients	Reports at 5 months: fatigue, sleep problems, concentration problems, and headache. Reports at 12 months: fatigue, sleeping problems, concentration problems, and headache
Tabacof et al., 2022 [152]	Retrospective cross-sectional observational study design	156 patients (all pre-vaccinations)	Most common long-lasting symptoms were fatigue, brain fog, and headache; most affected areas included self-care, anxiety/depression, and usual activities
Taquet et al., 2021 [153]	Cohort study	62,354 patients	Psychiatric diagnosis could be an independent risk factor for COVID-19
Taquet et al., 2021 [154]	Cohort study	236,379 patients	Significant neurological and psychiatric morbidity in the 6 months following COVID-19 infection recorded, particularly in severe COVID-19 cases
Tomasoni et al., 2021 [155]	Cross-sectional study	Patients with documented clinical recovery and virological clearance after hospitalization for COVID-19	29% of the patients suffered from anxiety; 11% of the patients suffered from depression
Wong et al., 2023 [78]	Cross-sectional study	2712 patients	Fatigue, cough, sore throat, difficulty concentrating, feeling of anxiety, myalgia, and arthralgia are the most common, severe long COVID symptoms
Xiao et al., 2022 [156]	Cross-sectional study	199 patients	Post-hospitalization and psychosocial factors showed a strong association with depression, anxiety, and post-traumatic growth
Zakia et al., 2023 [157]	Systematic review	23 articles	Anxiety, depression, sleep difficulties, and PTSD symptoms were prevalent among long COVID participants
Zawilska et al., 2022 [158]	Narrative review	-	Chronic fatigue, cognitive deficits, depression, anxiety, sleep disturbances, and sensory issues were common symptoms in long COVID
Pulmonary function and imaging			
Mandal et al., 2020 [159]	Cross-sectional study	384	Persisting symptoms and radiological abnormalities in a significant proportion of subjects
Taquet et al., 2021 [160]	Retrospective cohort study	273,618	Long COVID clinical features occurred and co-occurred frequently and showed some specificity to COVID-19, though they were also observed after influenza
Lewis et al., 2021 [161]	Retrospective analysis	80	No difference in pulmonary function was detected before and after COVID-19 infections in non-critically ill patients
Torres-Castro et al., 2021 [162]	Review and meta-analysis	380	Impaired lung function was observed in patients post-COVID-19 infection
Han et al., 2021 [163]	Follow-up study	114	Older age, acute respiratory distress syndrome, longer in-hospital stays, tachycardia, non-invasive mechanical ventilation, and higher initial chest CT scores are associated with the changes
Cellina et al., 2020 [164]	Pictorial essay	-	Imaging presentations of COVID-19 pneumonia are mostly GGO
Liao et al., 2023 [165]	Follow-up study	273	The most common CT findings were GGO and parenchymal bands at the 3- and 6-month follow-up sessions
Espallargas et al., 2021 [166]	Retrospective analysis	919	Pulmonary embolism in COVID-19 patients might predominantly affect segmental arteries and the right lung
Cardiological manifestations			
Xie et al., 2022 [167]	Cohort study	153,760	Risk and 1-year burden of cardiovascular disease in survivors of acute COVID-19 are substantial
Zuin et al., 2023 [168]	Meta-analysis	20,875,843	Myocarditis represents relatively rare, but important, post-acute COVID-19 sequelae
Lewek et al., 2021 [169]	Prospective cohort study	51	Severe cardiovascular complications in 27.5% of hospitalized patients
Ingul et al., 2022 [170]	Multicenter prospective cohort study and cross-sectional case–control study	204	Mild impairement of right and left ventricular functions at 3 months after discharge
Bhatia et al., 2023 [171]	Multicenter observational study	511	3% of athletes demonstrated de-novo ECG changes post-COVID-19 infection, of which 88% were diagnosed with cardiac inflammation
Kim et al., 2020 [172]	Observational study	510	Adverse RV remodeling predicts mortality in COVID-19 patients
Moody et al., 2021 [173]	Prospective cohort study	79	Ventricular remodeling in 29% of patients after 3 months
Małek et al., 2021 [174]	Retrospective cohort study	26	Cardiac abnormalities were found in CMR in a small group of athletes
Starekova et al., 2021 [175]	Prospective cohort study	145	Low prevalence of myocarditis (1.4%) among athletes recovering from COVID-19
Ammirati et al., 2020 [176]	Expert consensus document	-	Diagnosis and management of patients with myocarditis
Strain et al., 2022 [177]	Observational study	900	COVID-19 vaccination may help long COVID patients
Raman et al., 2022 [165]	Narrative review	-	Definition, epidemiology, and pathophysiology of cardiovascular manifestations of long COVID
Laranjera et al., 2022 [178]	Systematic review of longitudinal observational studies	5371	Electrocardiographic abnormalities indicative of myocarditis are uncommon in young athletes post-COVID-19

Legend: activities of daily living (ADLs); electroencephalogram (EEG); Magnetic Resonance Imaging (MRI); Montreal Cognitive Assessment (MoCA); quality of life (QoL); post-traumatic stress disorder (PTSD); liver function test (LFT); disorder of gut–brain interaction (DGBI); and post-infection functional gastrointestinal disorder (PI-FGID).

**Table 4:** Summary of the main studies on long COVID in pregnant women.

First Author, Year of Publication	Study Type	Population	Results
Sahin et al., 2021 [244]	Review	Pregnant women	SARS-CoV-2 infection can quickly lead to severe disease and higher rates of obstetric complications
Menges et al., 2021 [243]	Population-based cohort study	431 adults	A significant proportion of participants experienced longer-term consequences following SARS-CoV-2 infection
Muñoz-Chápuli Gutiérrez et al., 2024 [242]	Observational prospective study	409 pregnant women	34.2% of obstetric patients presented post-COVID-19 symptoms
Santos et al., 2022 [241]	Prospective cohort study	84 pregnant women	Pregnant women, mildly symptomatic, presented a greater risk of long-term complications, including ultrasound abnormalities, preterm birth, and postnatal depression
Oliveira et al., 2022 [240]	Longitudinal comparative study	588 pregnant women	The prevalence and duration of post-viral fatigue are higher in women infected during pregnancy, increasing with the severity of the infection
Afshar et al., 2020 [239]	Prospective cohort study	991 adults	COVID-19 has a prolonged and nonspecific disease course during pregnancy and in the 6 weeks after pregnancy
Kandemir et al., 2024 [238]	Single-center, cross-sectional, retrospective study	99 pregnant women	Long COVID prevalence was similar to the general population, correlating with severity, type, and number of symptoms of acute COVID-19
Vásconez-González et al., 2023 [237]	Cross-sectional comparative analysis	457 surveys	The most common long COVID symptoms were fatigue, hair loss, and a difficulty concentrating
Edlow et al., 2022 [236]	Retrospective cohort study	7772 live births, 7466 pregnancies, and 222 births to SARS-CoV-2-positive mothers	Maternal SARS-CoV-2 infection may be linked to neurodevelopmental sequelae in some offspring
Abbas-Hanif et al., 2022 [235]	Editorial	Adults	COVID-19 during pregnancy increases the risk of severe complications for both the mother and child
Villar et al., 2021 [234]	Multinational cohort study	2130 pregnant women	Consistent association between pregnant individuals with a COVID-19 diagnosis and higher rates of adverse outcomes
Machado et al., 2022 [233]	Editorial	Adults	Women with symptoms suggestive of long COVID should have the diagnosis confirmed and their health optimized before becoming pregnant
Sigfrid et al., 2021 [231]	Prospective, multicenter cohort study	327 hospitalized participants	Long-term symptoms, new disabilities, increased breathlessness, and reduced quality of life were noted in young females
Marchand et al., 2022 [229]	Systematic review and meta-analysis	754 COVID-19-positive pregnant women	Pregnant women who test positive for COVID-19 seem to be at a higher risk of lower birth weights and premature deliveries
Allotey et al., 2020 [228]	Systematic review	67,271 women	Pregnant and recently pregnant women with COVID-19 are more likely to be admitted to the intensive care unit or require invasive ventilation compared to non-pregnant women of a reproductive age
Metz et al., 2022 [230]	Retrospective cohort study	14,104 pregnant and postpartum patients	Among pregnant and postpartum women, SARS-CoV-2 infection was linked to a higher risk of a combined outcome of maternal mortality or severe morbidity due to obstetric complications

**Table 5:** Summary of main studies on long COVID in cancer patients.

Authors	Study Design	Population of Interest	Sample Size; Long COVID Population N (%)	Follow-Up Time	Age, Years	% Female Sex	Outcomes of Interest
Pinato et al., 2021 [246]	Registry study	COVID-19 cancer patients, solid or hematological malignances (active or in remission)	1557, 234 (15%)	44 (28–329) days	<65, 44.9% ≥65, 55.1%	45.5%	Post-COVID-19 sequelae symptom prevalence, risk factors
Cortellini et al., 2023 [247]	Registry study	COVID-19 cancer patients, solid or hematological malignances (active or in remission)	1909, 317 (16.6%)	39 (24–68) days	<65, 46.7% ≥65, 53.3%	54%	Post-COVID-19 sequelae symptom prevalence, risk factors in vaccinated and unvaccinated pts
Cortellini et al., 2022 [248]	Registry study	COVID-19 cancer patients, with the exclusion of advanced/metastatic malignances	186, 18 (9.8%) 100, 8 (8%)	6 months 12 months	<65, 45.2% ≥65, 54.8%	51.6%	Post-COVID-19 sequelae symptom prevalence at 6 and 12 months, risk factors
Lasagna et al., 2023 [249]	Telephone survey	COVID-19 cancer patients on active treatment, vaccinated with three doses of mRNA vaccines	97, 12 (12.4%)	12 weeks	58 (median)	91.7%	Post-COVID-19 sequelae symptom prevalence and data of patients treated with early anti-SARS-CoV-2 therapies
Dagher et al., 2023 [250]	Questionnaire	COVID-19 unvaccinated cancer patients, solid or hematological malignances (active or in remission)	312, 188 (60.2%)	14 days weekly for 3 months monthly up to 14 months	57, 21–86% <65, 63%, ≥65, 27%	63%	Post-COVID-19 sequelae symptom prevalence
Monroy-Iglesias et al., 2022 [251]	Telephone survey	COVID-19 cancer patiens, solid or hematological malignances	80, 41 (51.3%)	Symptoms occurred/worsened 4 weeks after COVID-19 diagnosis	<60, 36.6% >60, 61%	36.6%	Post-COVID-19 sequelae symptom prevalence, risk factors
Visentin et al., 2023 [252]	Retrospective multicenter study	COVID-19 patients with chronic lymphocytic leukemia	864, 137 (15.8%)	3 months 6 months	-	-	Overall and symptom prevalence, risk factors
Fankuchen et al., 2023 [253]	Cohort study	COVID-19 cancer patients with solid or hematological malignances and a matched cohort population	52, 15 (29%)	3 months 6 months 12 months	71, 61–83%	45%	Overall and symptom prevalence and post-discharge mortality
Hajjaji et al., 2022 [254]	Longitudinal online Survey	COVID-19 cancer patients with solid or hematological malignances (active or in remission)	2116, 37 (1.7%)	16 months	<60, 54% >60, 46%	81%	Prevalence of long-lasting symptoms in cancer patients with mild COVID-19 infection

## Data Availability

Not applicable.

## Supplementary Materials

The following supporting information can be downloaded at: https://www.mdpi.com/article/10.3390/diseases12050095/s1, Document S1: Multidisciplinary questionnaire for adults; Document S2: Multidisciplinary questionnaire for children.
