# Supplementary material for: Long COVID in Children, Adults, and Vulnerable Populations: A Comprehensive Overview for an Integrated Approach
Source: Diseases. 2024 May 6;12(5):95. doi: 10.3390/diseases12050095 (PMC11120262; doi:10.3390/diseases12050095)
Supplement: Supplementary file 1 [file diseases-12-00095-s001.zip › diseases-2969573-supplementary.pdf]

Supplementary Materials:

Document S1. Long-covid questionnaire for adults

ADULT PATIENTS

Patient description

Patient ID: ___________________________

Date of birth or age at the date of SARS-CoV-2 infection: ___________________________

Ethnic group: ☐ Arab ☐ Black ☐ Asian ☐ Latin American ☐ White ☐ Other: ____________________________

Sex: ☐ Female ☐ Male

If female, pregnancy:

☐ No

☐ Yes, before SARS-CoV-2 infection

☐ Yes, during SARS-CoV-2 infection (and other pregnancy before infection)

Date of SARS-CoV-2 infection: _ _ / _ _ / _ _ _ _

Vaccination status at the time of infection:

- Seasonal Influenza ☐ Yes ☐ No
- SARS-CoV-2 ☐ Yes ☐ No

If YES, please indicate the number of doses of SARS-CoV-2 vaccine: ☐ 0 ☐ 1 ☐ 2 ☐ 3 ☐ >3

Date of the last dose of SARS-CoV-2 vaccine: _ _ / _ _ / _ _ _ _

Symptomatic onset:

☐ No (incidental diagnosis)

☐ Yes (diagnosis following compatible symptoms)

Symptoms during the acute infection:

☐ Fever

☐ Cough

☐ Rhinorrhea

☐ Sore throat

☐ Fatigue

☐ Nausea

☐ Vomiting

☐ Diarrhea

☐ Myalgia

☐ Arthralgia

☐ Headache

☐ Conjunctival congestion

☐ Exertional dyspnea

☐ Dyspnea at rest

☐ Anosmia

☐ Skin manifestations

☐ Tachycardia

Admission:

☐ No

☐ Emergency Room
☐ Hospitalization in a department

Hospitalization:
☐ No
☐ Yes, no oxygen therapy needed
☐ Yes, oxygen therapy non- invasive ventilation required
☐ Yes, oxygen therapy invasive ventilation required

Medications:
☐ Corticosteroid
☐ Antiviral agents (Lopinavir-Ritonavir, Arbidol, Chloroquine phosphate, Hydroxychloroquine)
☐ Antibiotics
☐ Heparin

Comorbidities:
☐ Hypertension
☐ Diabetes
☐ Cardiovascular diseases
☐ Cerebrovascular diseases
☐ Malignant tumor in follow-up
☐ Malignant tumor in treatment
☐ COPD
☐ Chronic kidney disease
☐ Obesity
☐ Immunosuppression
☐ Psychiatric diseases

Weight at the date of infection: _____________ Kg

Height at the date of infection: _____________ m

Post COVID Syndrome

Respiratory problems: ☐ No ☐ Yes

If YES, which symptoms?
☐ Dyspnea
☐ Cough
☐ Sore throat

Possible responses:
☐ No, never
☐ No, symptom resolved
☐ Yes, even before infection
☐ Yes, after acute infection

Fatigue? ☐ No ☐ Yes

If YES, which problems?
☐ Fatigue/deep general tiredness
☐ Tiredness in activities of daily living

Any problems in movements of limbs and face? ☐ No ☐ Yes

If YES, which problems?

- ☐ Slowing down in the execution of movements
- ☐ Tremor/involuntary movements of limbs
- ☐ Weakness/inability to move limbs (> 1 hour)
- ☐ Loss of sensibility/ tingles/ burning in the limbs (> 1 hour)
- ☐ Walking problems
- ☐ Vertigo
- ☐ Complete or partial facial paralysis
- ☐ Urinal/ fecal incontinence

Myalgia/ joints pain/ chest pain/ headache/ facial pain? ☐ No ☐ Yes

If YES, which ones?

- ☐ Severe myalgia
- ☐ Arthromyalgia
- ☐ Chest pain
- ☐ Facial pain
- ☐ Headache

Any problems with taste/ smell/ vision or dizziness? ☐ No ☐ Yes

If YES, which problems involving sense organs?

- ☐ Alteration of taste
- ☐ Alteration of smell
- ☐ Diplopia (> 1 hour)
- ☐ Blurred vision (> 1 hour)

Loss of consciousness/ brain fog/ cognitive problems? ☐ No ☐ Yes

If YES, which neurological/ cognitive disorders?

- ☐ Loss of consciousness
- ☐ Loss of contact with your surrounding environment
- ☐ Problems speaking/ understanding what people are saying to you
- ☐ Problems with orientation outside your home
- ☐ Trouble remembering what day it is
- ☐ Trouble remembering the names of relatives and friends
- ☐ Episodes of uncontrolled laughter or crying
- ☐ Irritability/ reactivity towards others

Any sleep problems? ☐ No ☐ Yes

If YES, which ones?

- ☐ Insomnia
- ☐ Poor sleep quality
- ☐ Daytime sleepiness
- ☐ Night sweats

Any psychiatric symptoms? ☐ No ☐ Yes

If YES, which ones?

- ☐ Anxiety symptoms

☐ Mood deflection
☐ Suicidal thoughts
☐ Flash back
☐ Obsession and/or compulsions

Any gastrointestinal symptoms? ☐ No ☐ Yes

If YES, which symptoms?
☐ Weight loss
☐ Diarrhea
☐ Gastric pain
☐ Abdominal pain
☐ Constipation

Dermatological diseases? ☐ No ☐ Yes

Do you follow a Long-COVID rehabilitation program? ☐ No ☐ Yes

Document S2. Long-covid questionnaire for children

PEDIATRIC PATIENT

Demographic data
Patient ID: ________________________
Sex: ☐ Male ☐ Female
Date of birth: _ _ / _ _ / _ _ _ _
Ethnicity: ☐ Arab ☐ Black ☐ Asiatic ☐ Latin American ☐ White ☐ Other: ________________________

Amnestic data
Birth weight: ____________ Kg
Gestational age: ________ weeks + ____ days

Delivery: ☐ Vaginal ☐ Caesarean

Risk factors in pregnancy:

Mother
☐ Age:_____ years
☐ Diabetes
☐ Obesity
☐ Smoking
☐ Alcohol consumption
☐ Autoimmune diseases
☐ Stress
☐ Sedentary lifestyle
☐ Other: ___________________________________

Father
☐ Age:______ years
☐ Smoking
☐ Alcohol consumption

Breastfeed? ☐Yes ☐No ☐N/A

Weaning period: _____ months

SARS-CoV-2 infection data
Date of SARS-CoV-2 infection: _ _ / _ _ / _ _ _ _
Variant (if known): _________________________
Admission in: ☐ Pediatric Unit ☐ ICU

Vaccination

Vaccinations appropriate for age ☐ Yes ☐ No

- Seasonal Influenza ☐ Yes ☐ No
- SARS- CoV-2 ☐ Yes ☐ No

If YES, please indicate the number of doses of SARS- CoV-2 vaccine: ☐ 1 ☐ 2 ☐ 3 ☐ >3

Pre-existing comorbidities

☐ Chronic heart disease (including congenital heart disease)
☐ Obesity
☐ Diabetes
☐ Asthma
☐ Chronic renal disease
☐ Rheumatological disease
☐ Chronic bowel disease
☐ Liver disease
☐ Neuropsychiatric disorder
☐ Neurological disease
☐ Malnutrition
☐ Hematologic disease
☐ Immunodeficiency
☐ Other immunological disorders
☐ Malignant neoplasm
☐ Genetic diseases
☐ Endocrinological diseases
☐ Other: ____

Long-COVID symptoms

Does your son/daughter have symptoms/disorders after COVID-19? ☐ Yes ☐ No

Which of these symptoms/disorders has your child had up to date?

☐ Fever
☐ Fatigue
☐ Inappetence
☐ Nasal congestion/ rhinitis
☐ Sore throat
☐ Wheezing
☐ Cough
☐ Dyspnea
☐ Tachycardia
☐ Chest tightness
☐ Chest pain
☐ Myalgia
☐ Joints pain and/or swelling
☐ Headache
☐ Dizziness
☐ Mood disorders
☐ Cognitive disorders
☐ Difficulty concentrating
☐ Alteration of smell
☐ Alteration of taste
☐ Sleep problems
☐ Increased need for sleep
☐ Sensory/ motor symptoms
☐ Weight loss

☐ Diarrhea
☐ Gastric pain
☐ Abdominal pain
☐ Constipation
☐ Skin rashes/ lesions
☐ Other: ____

Since the symptoms have been reported, how long did your child have them? _________________________________

Has your son/daughter been unable to go to school because of these symptoms? ☐Yes ☐No

Have your child taken/is your child taking medications to treat these symptoms? ☐Yes ☐No

If YES, specify which medications: ____
